# Cardiac remodelling and dysfunction in cancer patients receiving cardiotoxic therapies: proteomic and metabolomic profiling

**DOI:** 10.1093/eurheartj/ehag487

**Published:** 2026-09-16

**Authors:** Bonnie Ky, Congying Xia, Kyunga Ko, Craig Hyde, Amanda M. Smith, June-Wha Rhee, Cheryl Tow-Keogh, Cassandra Tierney, Tao Long, Liyong Zhang, Peter P. Liu, Nicholas S. Wilcox, Vinh Dang, Saro H. Armenian, Mohit Jain, Raja Mangipudy, Vishal S. Vaidya

**Affiliations:** 1 Division of Cardiology, Department of Medicine, Perelman School of Medicine, University of Pennsylvania, Philadelphia, PA 19104, USA;; 2 Thalheimer Center for Cardio-Oncology, Abramson Cancer Center, Perelman School of Medicine at the University of Pennsylvania, Philadelphia, PA 19104, USA;; 3 Department of Biostatistics, Epidemiology and Informatics, Perelman School of Medicine, University of Pennsylvania, Philadelphia, PA 19104, USA;; 4 Research and Development, Pfizer Inc, 1 Portland Street, Cambridge, MA 02139, USA;; 5 Division of Cardiology, Department of Medicine, City of Hope Comprehensive Cancer Center, Duarte, CA 91010, USA;; 6 Sapient Bioanalytics LLC, San Diego, CA 92121, USA;; 7 University of Ottawa Heart Institute, University of Ottawa, Ottawa, Ontario, K1N 6N5, Canada;; 8 Department of Population Sciences, City of Hope Comprehensive Cancer Center, Duarte, CA 91010, USA

**Keywords:** Cardiotoxicity, Breast cancer, Biomarkers, Proteomics, Metabolomics

## Abstract

**Background and Aims:**

The objective of this study was to define the relationships between the circulating proteome and metabolome with cardiac structure and function in patients with breast cancer receiving cardiotoxic therapies.

**Methods:**

Proteomics and metabolomics profiling was performed in a longitudinal, prospective cohort study of breast cancer patients receiving anthracyclines and/or trastuzumab, using the Olink Explore 3072 platform and rapid liquid chromatography-mass spectrometry, respectively. Multivariable linear mixed-effect models evaluated the contemporaneous (same visit) and lagged (subsequent visit) associations between repeated measures of individual proteins or metabolites with quantitative echocardiographic measures of cardiac structure [left ventricular (LV) mass and left atrial volume index] and function [LV ejection fraction (LVEF), longitudinal and circumferential strain, *E*/*e*′, and ventricular-arterial coupling]. Cox regression and pathway enrichment analyses were conducted for biomarkers demonstrating significant associations with cardiac function.

**Results:**

Across 547 breast cancer participants (median age 50 years), 203 unique proteins and 16 unique metabolites were significantly associated with measures of cardiac structure and function in contemporaneous and lagged analyses. Notably, cathepsin C was associated with LVEF [false discovery rate (FDR), *P* = .017], longitudinal strain (FDR, *P* = .046), left atrial volume index (FDR, *P* = .035), and incident cardiac dysfunction, defined by an LVEF decline ≥10% to <50% (hazard ratio .61, 95% confidence interval .41, .90). The 147 proteins associated with cardiac function were enriched in biological processes reflective of protein deubiquitination, protein modification by small protein removal, macromolecule catabolic processes, and global metabolic pathways. Individual metabolites significantly associated with cardiac function (LVEF, longitudinal strain) included *n*-acetylglutamine, aspartic acid, acetylasparagine, alanyl-alanine, and prolyl-glycine (FDR, *P*-value < .001), and belonged to amino acids and derivatives and peptides.

**Conclusions:**

These findings provide translational insights into cancer therapy-related cardiac dysfunction and remodelling and identify potential new biomarkers of cardiotoxicity. There is an important need for validation of these findings and a deeper understanding of the biology of these biomarkers.

## Graphical Abstract

**Key Question:**

How is the proteomic and metabolomic profile associated with cardiac remodelling and dysfunction in patients with breast cancer receiving cardiotoxic therapies?

**Key Finding:**

In a cohort of breast cancer patients, cathepsin C, aspartic acid, acetylasparagine, n-acetylglutamine, alanyl-alanine, and prolyl-glycine were among the 203 unique proteins and 16 metabolites significantly associated with multiple measures of cardiac remodelling and dysfunction.

**Take Home Message:**

These findings provide new translational insights into the biological landscape of cancer therapy-related cardiac dysfunction.

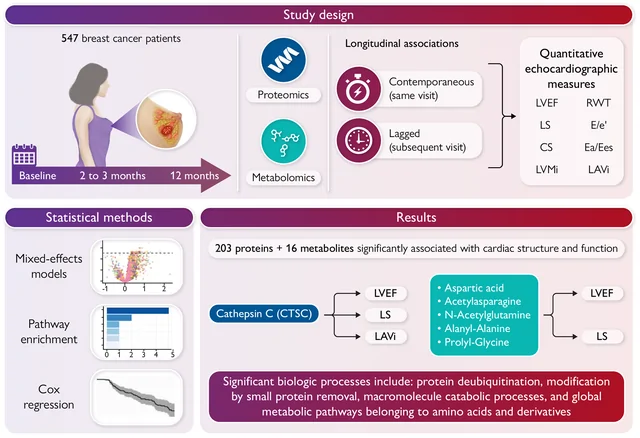

**CS**, circumferential strain; **Ea/Ees**, ventricular–arterial coupling; **E/e’**, ratio of early mitral in.ow velocity to early diastolic mitral annular velocity; **LAVi**, left atrial volume index; **LS**, longitudinal strain; **LVEF**, left ventricular ejection fraction; **LVMi**, left ventricular mass index; **RWT**, relative wall thickness

Proteomics and Metabolomics Profiling in Patients with Breast Cancer Receiving Cardiotoxic Therapies.

## Introduction

Cardiovascular disease and cancer are two leading causes of morbidity and mortality worldwide.^1^ It is well established that cancer patients are at increased risk of cardiovascular disease.^2^ In breast cancer specifically, the most common female malignancy worldwide, patients receive several potentially cardiotoxic therapies including anthracyclines, HER2+ targeted, radiation, and hormone therapies.^3–5^ Moreover, cancer therapy-related cardiotoxicity is influenced by patient-specific risk factors, including age, cardiovascular risk factor burden (hypertension, dyslipidaemia, diabetes, obesity), and presence of cardiovascular disease (coronary artery disease, heart failure, arrhythmia, stroke).^6^ However, to date, there is a limited understanding of the underlying biologic perturbations that occur with these cancer therapies, as well as the translation of this knowledge to the subsequent identification of patients at risk for cardiac dysfunction and adverse remodelling.

Established circulating biomarkers that are sensitive and specific for cardiotoxicity risk remain limited and represent a significant limitation for the field of cardio-oncology.^7^ Current markers largely lack the discriminative accuracy to diagnose cancer therapy-related cardiotoxicity and identify patients at increased cardiovascular risk. For example, clinical biomarkers of cardiac injury, such as troponin, are non-specific and are not predictive of cardiac dysfunction.^8,9^ Discovery of new circulating markers that can provide insights into the pathophysiologic changes in cardiac structure, function, and remodelling that occur following cancer therapy is a necessary first step towards improved cardiotoxicity risk prediction.

Proteomics-based high-throughput technologies and metabolomics analyses using liquid chromatography–mass spectrometry have led to the identification of potential biomarkers and signalling pathways in cancer and cardiovascular disease, and hold promise for the identification of novel biomarkers of cardiotoxicity in cardio-oncology.^10–12^ However, to date, only a few human studies have applied these approaches to investigate potential biomarkers for cardiotoxicity. Findings have been variable across these studies, which may relate to several factors, including modest sample size, heterogeneity in study population characteristics (e.g. cancer type, treatment regimen, and time since cancer therapy exposure), and differences in analytic approaches and the exact question of interest.^12–16^ It remains unknown how differences in proteomic or metabolomic profiles contribute to cardiac remodelling and dysfunction, and if they can ultimately be used to identify patients with an increased susceptibility to cancer therapy-related cardiotoxicity. To address these knowledge gaps, we quantified the longitudinal changes in circulating proteomic and metabolomic markers and determined their associations with comprehensive centrally quantified measures of cardiac function and remodelling, including left ventricular (LV) structure (LV mass index, relative wall thickness) and systolic (LVEF, longitudinal and circumferential strain, ventricular–arterial coupling) and diastolic function [left atrial (LA) volume index and *E*/*e*′] in a longitudinal cohort of patients with breast cancer.

## Methods

### Study population

The Penn Cardiotoxicity of Cancer Therapy Cohort Study (NCT01173341) is a longitudinal, prospective cohort study of breast cancer patients recruited from multiple sites across the University of Pennsylvania Health System Abramson Cancer Center.^17^ Patients aged at least 18 years, diagnosed with breast cancer, and treated with doxorubicin and/or trastuzumab, with or without regional nodal photon or proton radiation therapy, were enrolled. For all participants, cancer therapy regimens were determined by the treating oncologist and consisted of doxorubicin (240 mg/m^2^ divided into four cycles of 60 mg/m^2^ each) and cyclophosphamide followed by paclitaxel (Dox), or doxorubicin (240 mg/m^2^ divided into four cycles) and cyclophosphamide followed by paclitaxel and trastuzumab (Dox + Tras), or trastuzumab with docetaxel and either cyclophosphamide or carboplatin (Tras), but not doxorubicin.

All studies were approved by the Institutional Review Board of the University of Pennsylvania. All participants provided written informed consent prior to study enrolment.

### Study procedures

The longitudinal cohort study design has been previously published.^17^ Study visits occurred at baseline (prior to cancer therapy initiation) and at subsequent standardized time intervals during and after cancer therapy completion. At each study timepoint, a blood sample, an echocardiogram, and a patient-administered questionnaire were obtained on/near the same day.

Clinical data included demographics (age, race), cardiovascular risk factors (hypertension, hyperlipidaemia, tobacco use, family history of cardiac disease), cardiovascular disease (arrhythmia, heart failure, coronary disease, stroke), measured variables (blood pressure, weight, height), cancer history (stage, hormone status), treatment regimens (anthracyclines, Her2+ targeted, radiation, hormone therapies), and cardiovascular medications (angiotensin-converting enzyme inhibitors, angiotensin receptor blockers, beta blockers, sodium-glucose cotransporter 2 inhibitors, diuretics, neprilysin inhibitors, lipid-lowering, anti-platelet or anticoagulant therapies). These were obtained through standardized questionnaires and medical record review.

Plasma samples were collected in EDTA tubes and stored at −80°C. Samples obtained at baseline and during prespecified intervals, including 2–3 months and 12 months post-cancer therapy initiation, were used for proteomics and metabolomics profiling (see Supplementary data online, Figure S1).

Echocardiograms were performed at Intersocietal Accreditation Commission laboratories using Vivid 7, E9, or E95 machines (GE Healthcare) according to a standardized echocardiography imaging protocol. Transthoracic echocardiograms were performed in the Dox group at baseline, at the completion of paclitaxel chemotherapy (~4 months), and then annually until Year 5 and then biennially. In the Dox + Tras group, echocardiograms were performed at baseline, at the completion of doxorubicin (~2 months), every 3 months during trastuzumab therapy, and then annually until Year 5 and then biennially. In the Tras group, echocardiograms were performed at baseline, every 3 months during trastuzumab therapy, and then annually until Year 5 and then biennially. For the purposes of this analysis, we focused on echocardiograms performed up to 2 years after cancer therapy initiation, as our main question of interest related to the diagnosis and prediction of early/subacute cardiotoxicity, and we hypothesized that the relevance of these biomarkers to cardiac remodelling and dysfunction could change over time. Moreover, previously published data support this time frame as that with the greatest cardiotoxicity incidence.^18,19^

### Proteomics analysis

Plasma samples were examined using the Olink Explore 3072 platform using the Proximity Extension Assay (PEA) technology and next-generation sequencing (NGS). The PEA technology uses matching pairs of oligonucleotide-labelled antibody probes that bind to the target antigens, producing a binding complex of complementary oligonucleotides close to each other, resulting in target sequence formation. In the Olink Explore protocol, the target sequence was amplified in a double PCR reaction and purified before the NGS. The sequence data were then processed and normalized to produce Olinks’ relative quantification unit Normalized Protein eXpression (NPX).^20^ Sample processing and library preparation were performed according to the manufacturers’ guidelines (Olink User Manual ver. 1191.v3.1.2023-.6-26). After the post-cleanup, purified libraries underwent quality control with the High-Sensitivity D1000 kit on the Agilent 4200 Tapestation. The pooled libraries were then sequenced on an Illumina NovaSeq6000 at the Olink Service Laboratory in Waltham, MA, USA. The initial quality control was performed using the Olink NPX software (Ver 1.10.0-1-x-Support11).

### Metabolomics analysis

The plasma samples (stored at −80°C) were randomized and thawed over 10 min using a custom rapid plate thaw system, then placed on an orbital shaker at 1250 rpm at 4°C for 10 min. The plasma samples were processed as per the procedure described in the Supplementary data online, Material. The plasma was transferred into 200 μL of an acidified solution of acetonitrile and methanol in a shallow 96-well microtiter plate with internal standards (13C515N1-Glutamic acid [Sigma-Aldrich], CUDA [Cayman Chemicals], 16:0-d31-18:1 PC [Avanti], 14:0-16:1-14:0-d5 TG [Avanti], and arachidonic acid-d11 [Cayman Chemicals]). Samples were shaken at 550 rpm at 4°C for 10 min, followed by centrifugation at 6000 *g* at 4°C for 10 min. For positive ion mode, 2 μL of supernatant was added to 148 μL of water:methanol (80:20) containing .05% formic acid, 1 mM ammonium formate, and internal standards MAPCHO-12-d38 (Avanti Lipids), Phenylalanine-d5 (Sigma-Aldrich). For negative ion mode, 15 μL of supernatant was added to 135 μL of water:methanol (60:40) containing .1% acetic acid and internal standards MAPCHO-12-d38 (Avanti Lipids), Phenylalanine-d5 (Sigma-Aldrich). All samples were de-identified before receipt, and all analyses were completed in a blinded fashion (Sapient Bioanalytics, San Diego, CA, USA).

### Rapid liquid chromatography–mass spectrometry

Samples were analysed by rapid liquid chromatography–mass spectrometry (rLC–MS)^21^ and were injected onto a custom-packed silica-based mixed-mode column that allows for both reverse-phase and weak ion-pairing retention mechanisms. The sample analysis, packing, and data acquisition are detailed in the Supplementary data online, Methods. A separate aliquot of pooled commercial EDTA plasma (BioIVT, Westbury, NY, USA) was also extracted and injected as an external QC sample. Rigorous data QC (Supplementary data online, Methods) was performed using the panel of isotopically labelled internal standards and interval-pooled plasma samples to monitor fluctuations in extraction efficiency, instrument sensitivity, matrix artefact, and mass accuracy. Mass calibrations (passing threshold of 1 ppm) were performed before each 384-well plate run to assess mass accuracy, mass resolution, detector sensitivity, and instrument cleanliness.

### Spectral data handling, extraction, and alignment

Raw data were converted to mzXML using MSConvert (Proteowizard 3.0). Chromatographic drift was assessed and corrected based on common landmarks observed in all samples.^22^ Metabolite features were extracted from drift-corrected mzXML files using custom imaging processing-based software.^23^ After data extraction, molecular features were normalized to account for plate-to-plate variation using a batch median normalization metric with correction for median levels. Compound identification was performed by searching collected data against Sapient’s internal commercial standard compound library.

### Quantitative echocardiography

Quantitative echocardiography was performed at the Penn Center for Quantitative Echocardiography by trained sonographers blinded to participant characteristics, using the TomTec Imaging Systems software and according to the American Society of Echocardiography guidelines^24^ if specified, and according to previously published, established methodologies.^18,25^ This included quantification of *LV systolic function* [LVEF, strain (longitudinal, circumferential)], *LV structure* [LV mass indexed to body surface area (BSA), relative wall thickness], *LV diastolic function* [*E*/*e*′ (early diastolic mitral inflow velocity to early diastolic mitral annulus velocity), LA volume indexed to BSA], and *ventricular-arterial (VA) coupling* (Ea/Ees; where Ea indicates effective arterial elastance, and Ees the slope of the end-systolic pressure-volume relation).^18,25^ Cardiac dysfunction was defined as an LVEF decline ≥10% from baseline to <50%. LVEF was calculated using Simpson’s method of discs.^24^ Strain (%) was reported in absolute values.^26^ The intra-observer coefficient of variation for LVEF was 4.4%, longitudinal strain 10.9%, and circumferential strain 9.4%. The intra-observer coefficient of variation for mitral inflow and tissue Doppler velocities were 2.3%–5.4%, and for the Doppler timing intervals used to derive Ees were .3%–5.7%.^18^

### Statistical analysis

#### Associations between proteomic/metabolomic biomarkers and cardiac remodelling and function

Supplementary data online, Figure S1 details our analytic approach. Standard descriptive statistics were used to summarize participant characteristics at baseline, including median (Quartile [Q]1, Q3) for continuous variables and counts (percentages) for categorical variables. Graphical displays including the trajectory of each echocardiographic measure over time were evaluated using Locally Estimated Scatterplot Smoothing (LOESS) curves with 95% confidence interval (95%CI).

Each biomarker (protein or metabolite) was examined individually in linear mixed-effect models to determine the association between biomarker and quantitative echocardiographic measure of interest (LVEF, longitudinal strain, circumferential strain, LV mass index, relative wall thickness, *E*/*e*′, LA volume index, VA coupling). These echocardiographic measures were a priori selected given their prognostic significance.^18,27^ Models included a random intercept for each participant to account for within-subject correlation and covariates adjusted as fixed effects. These covariates included age, race (Black, White), cancer treatment (Dox, Tras, Dox + Tras), body mass index, smoking status (never, former, current), hypertension, diabetes, visit (baseline, 2–3 months, 12 months), and time interval between echocardiography acquisition and blood sample. We first investigated ‘contemporaneous’ associations between biomarkers and echocardiographic measures by fitting separate models using the exposure (proteome/metabolome) and outcome (each of the eight quantitative echocardiographic measures) obtained at the *same* visit, specifically at baseline, 2–3 months, and 12 months post-cancer therapy initiation. We considered these ‘diagnostic’ biomarkers. ‘Lagged’ associations between biomarkers and echocardiographic measures were then evaluated using echocardiography measures obtained at the immediate visit, *subsequent* to the blood sample, indicative of ‘predictive’ biomarkers. All analyses were corrected for multiple testing using the Benjamini and Hochberg procedure. Within each of these models, biomarkers were considered significant if the false discovery rate (FDR) was <.05.

To explore whether significant associations between biomarkers and echocardiographic measures differed across cancer treatment groups, biomarker-treatment interaction terms were introduced in sensitivity analyses. We also explored whether these associations differed by race (Black, White). Statistical significance was assessed using Wald tests, and the significance threshold was corrected for multiple testing with the Bonferroni method.

Biomarkers demonstrating significant associations (FDR <.05) with measures of cardiac function (LVEF, longitudinal strain) in the contemporaneous or lagged analyses were then combined into a union set and consequently used for additional exploratory analyses. Significant biomarkers were also used to determine the multivariable associations between baseline concentrations and incident cardiac dysfunction using penalized Cox proportional hazard models with least absolute shrinkage and selection operator (LASSO). Ten-fold cross-validation of the model was performed to optimize the hyper-parameter. The protein that was selected through LASSO was subsequently entered into a standard Cox regression for estimation of the hazard ratio and 95%CI. The Cox regression model was adjusted for age, race (Black, White), cancer treatment (Dox, Tras, Dox + Tras), body mass index, smoking status (never, former, current), hypertension, and diabetes. Correlations between significant biomarkers and conventional measures of high-sensitivity troponin T (hsTnT) and NTproBNP^17^ were also performed using Pearson correlation, and Cox models were further adjusted for these two markers. To explore whether biomarkers improved prediction of cardiac dysfunction and to gain insights into their potential clinical utility, a number of analyses were performed. Baseline biomarker values (on the NPX scale) were added to a clinical risk factors only model, as were the conventional biomarkers, NTproBNP and hsTnT. This clinical risk factors only model, which served as the reference, was based on covariates used in the aforementioned multivariable association Cox models, selected for consistency and included: age, race, cancer treatment, body mass index, smoking status, hypertension, and diabetes. The concordance index and time-dependent area under the receiver operating characteristic curve (AUC)^28,29^ and 95%CI at 6 and 12 months were derived for each of these models, as was the integrated discrimination index (IDI) and category-free net reclassification index (cNRI).^30,31^ The variance of the concordance index was estimated using the infinitesimal jack-knife method implemented in the R ‘survival’ package, and the 95%CI was then estimated using a normal approximation. Time-dependent AUC and 95%CI were estimated using inverse probability of censoring weighting approach (R ‘timeROC’ package). CI of the IDI and cNRI was derived based on perturbation resampling, implemented in the R ‘survIDINRI’ package. All analyses were conducted using R statistical Software v4.4.0 (Foundation for Statistical Computing, Vienna, Austria).

#### Pathway enrichment analyses and proteomic/metabolomic biomarker correlations

Biomarkers noted above in our exploratory analysis also served as a candidate set of markers for pathway enrichment analysis. Separate pathway enrichment analyses were performed for the sets of proteins showing positive vs negative associations. The background list was defined as proteins measured in this study and that passed quality control. Gene Ontology (GO) enrichment was assessed for biological process, cellular component, and molecular function. We also explored pathway enrichment analyses based on the Kyoto Encyclopedia of Genes and Genomes (KEGG) pathway database (https://rest.kegg.jp). Enrichment was tested using Fisher’s exact test, and *P*-values were adjusted for multiple testing using the FDR procedures. Analyses were conducted using the R ‘clusterProfiler’ package.

Moreover, pairwise correlations between proteomic and metabolomic biomarkers were explored at each of the three timepoints (baseline, 2–3 months, 12 months) using Pearson’s method. Protein–metabolite pairs that consistently show an absolute correlation magnitude of .3 or greater were considered potentially important.

## Results

### Participant characteristics

Among the 547 participants, the median age was 50 years (Q1, Q3 41, 58), and 26.3% were Black (Table 1). In terms of cancer characteristics, 53.2% had Stage 2 disease, and 48.0% had left-sided breast cancer. Overall, 60.7% received Dox, 14.4% received Dox + Tras, 24.9% received Tras, and 35.0% underwent left-sided radiation therapy. At baseline, prior to the initiation of cancer therapy, 31.1% had hypertension, 25.2% hyperlipidaemia, and 9.0% diabetes, with 40.7% being treated with a cardiovascular medication. The baseline prevalence of cardiovascular disease, as defined by heart failure, coronary disease, arrhythmia, or stroke, was 10.2%.

### Echocardiographic measures of cardiac structure and function

**Figure 1:**
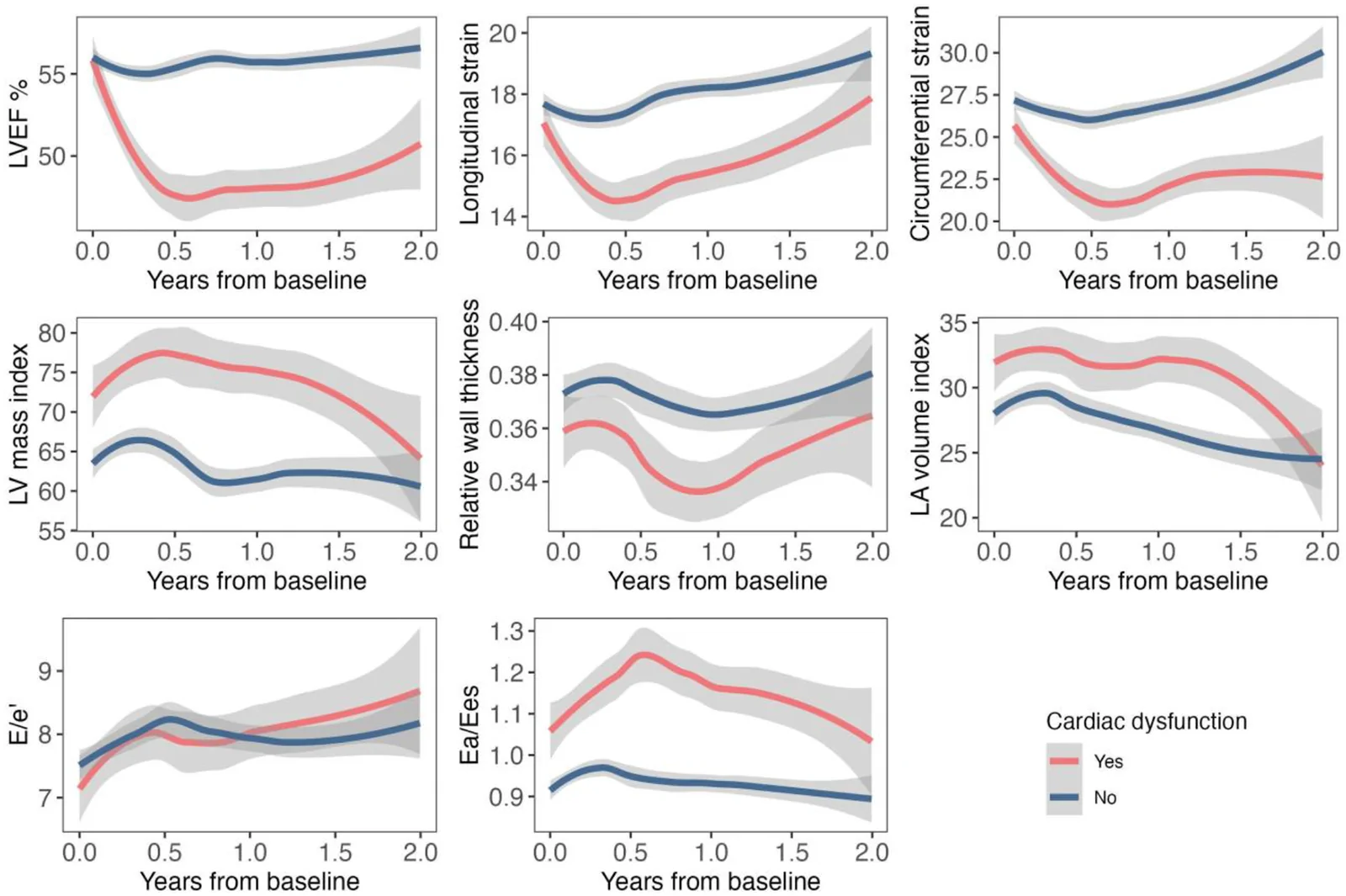
Trajectories of echocardiographic measures over time. Loess smoothing splines and 95% confidence interval for echocardiographic measures according to cardiac dysfunction. Cardiac dysfunction was defined by a ≥ 10% decline in LVEF to a value <50%. Red indicates participants who experienced cardiac dysfunction; blue indicates participants who did not experience cardiac dysfunction. Longitudinal and circumferential strains are reported in absolute values. LVEF, left ventricular ejection fraction; LV, left ventricular; LA, left atrial

At baseline, quantitative echocardiographic measures of cardiac structure and function were normal. Trajectories over time are shown in Figure 1, and demonstrate that on average, there was a modest, early worsening of cardiac systolic and diastolic function. Overall, 14% experienced cardiac dysfunction, as defined by a ≥ 10% decline in LVEF to a value <50%, over a median time of 7 months (Q1, Q3 4, 12 months) (see Supplementary data online, Figure S2).

### Associations between proteomics and cardiac structure and function

**Figure 2:**
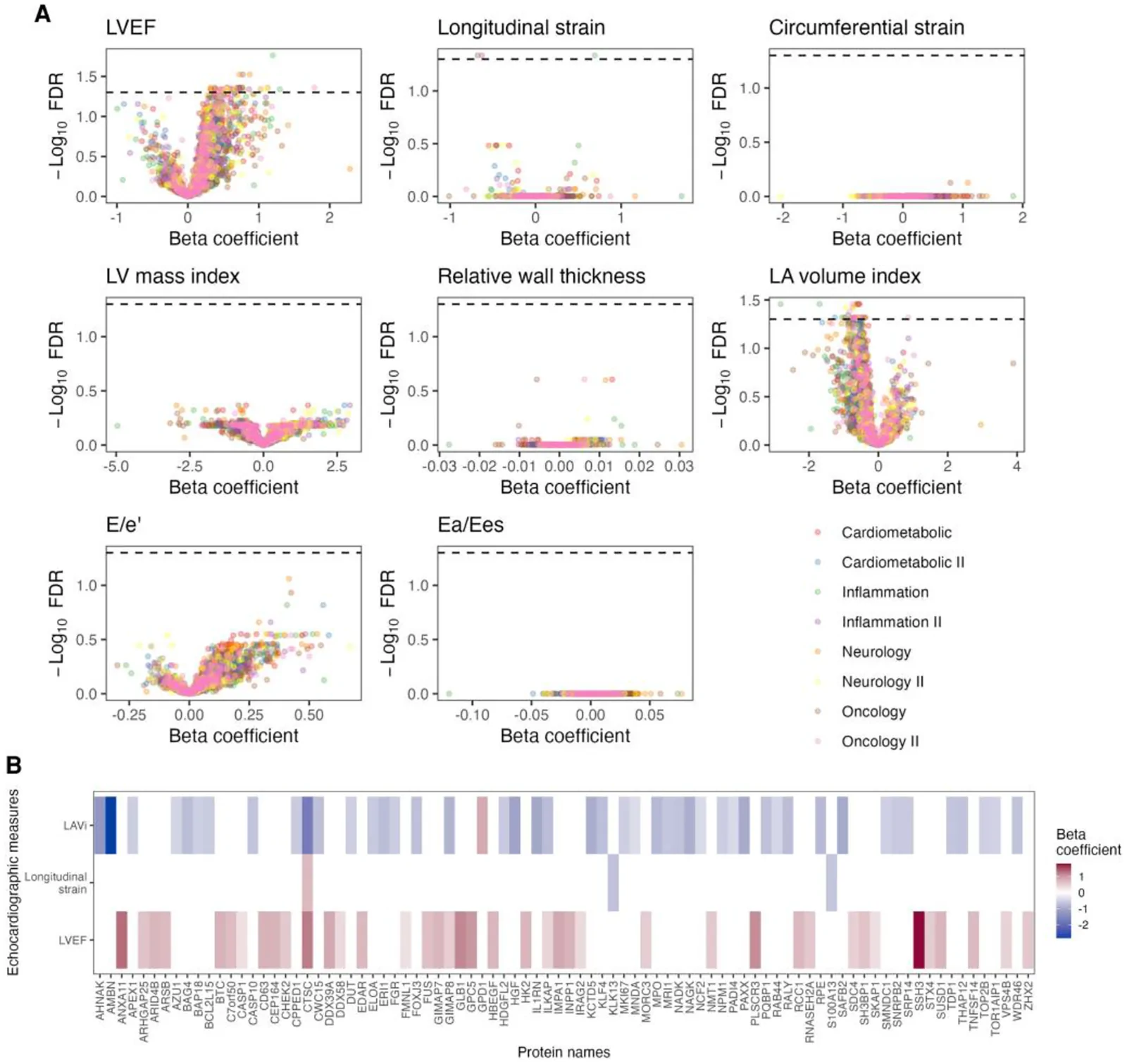
(*A*) Volcano plot for the contemporaneous associations between all proteins and cardiac structure and function. Contemporaneous associations between proteomic and echocardiographic measures were evaluated using measures obtained at the same visit. Linear mixed-effect models were adjusted for age, race (Black, White), cancer treatment (Dox, Tras, Dox + Tras), body mass index, smoking status, hypertension, diabetes, visit (baseline, 2–3 months, 12 months), and time interval between echocardiography acquisition and blood sample. P-values were corrected for multiple testing using the Benjamini and Hochberg procedure. Dashed lines indicate the significance level of FDR .05. Dots are colour-coded according to Olink panel. LVEF, left ventricular ejection fraction; LV, left ventricular; LA, left atrial. (*B*) Magnitude of the associations for proteins that were significant in the contemporaneous analysis. Heat map of the magnitude of beta coefficients for significant proteins derived from the linear mixed-effect models. Red indicates positive associations, and blue indicates negative associations, with a darker colour indicating a larger absolute value. Ameloblastin (AMBN) and cytoplasmic glycerol-3-phosphate dehydrogenase (GPD1) had the strongest association with left atrial volume index in terms of magnitude (negative and positive association, respectively). Protein phosphatase Slingshot homolog 3 (SSH3) had the strongest positive association with LVEF. Kallikrein-13 (KLK13) and Cathepsin C (CTSC, also known as Dipeptidyl peptidase 1) had the strongest association with longitudinal strain in terms of magnitude (negative and positive association, respectively). LVEF, left ventricular ejection fraction; LAVi, left atrial volume index

First, we determined the adjusted associations between the circulating proteome and echocardiographic measures at the same visit in our contemporaneous analysis. The median time between blood sampling and echocardiography across all three timepoints was 0 (Q1, Q3 0, 11 days). After FDR correction, there were 40 proteins that showed significant associations with LVEF; three with longitudinal strain, and 47 with LA volume index (Figure 2A and B, Supplementary data online, Table S1). Of note, cathepsin C (CTSC) was significant across all three echocardiographic measures [LVEF (FDR, *P* = .017), longitudinal strain (FDR, *P* = .046), LA volume index (FDR, *P* = .035)], and of all the proteins associated with these measures, demonstrated some of the strongest effect sizes. In the same cohort, baseline levels of CTSC were also significantly associated with risk of cardiac dysfunction in our multivariable Cox regression analyses (hazard ratio .61, 95%CI .41, .90, per 1-unit NPX change); this association remained significant with adjustment for conventional biomarkers hsTnT and NTproBNP (hazard ratio .62, 95% CI .42, .91, per 1-unit NPX change).^17^ In exploratory analyses, addition of baseline CTSC to a model including clinical variables alone yielded a modest numerical increase in the concordance index from .66 to .68, and in the time-dependent AUC from .64 (95% CI .55, .73) to .68 (95% CI .59, .77) at 6 months, with similar findings at 12 months (see Supplementary data online, Table S2A). In evaluation of risk reclassification metrics, there was a similarly modest increase in both the IDI and cNRI at 6 (IDI .006, cNRI .154) and 12 months (IDI .014, cNRI .104) (see Supplementary data online, Table S2B). In comparison, biomarkers NTproBNP and hsTnT yielded non-significant hazard ratios (see Supplementary data online, Table S2C) and non-significant differences that were of lesser magnitude for measures of discrimination and reclassification. S100A13 was also notable as having one of the strongest negative associations with longitudinal strain (*b* −.63, FDR *P* = .046). There were no proteomic markers that were associated with circumferential strain, Ea/Ees, *E*/*e*′, LV mass index, or relative wall thickness.

**Figure 3:**
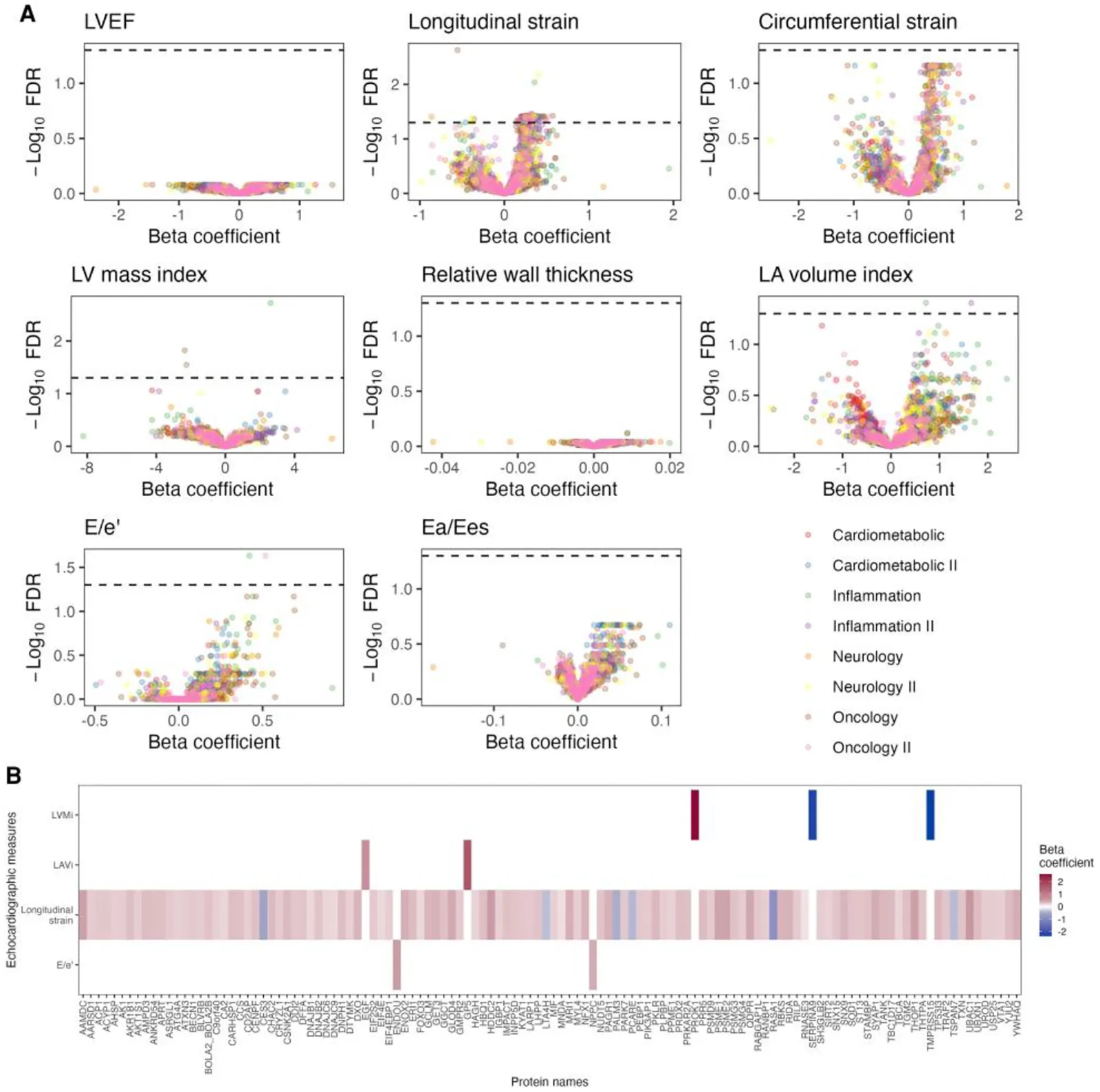
(*A*) Volcano plot for the lagged associations between all proteins and cardiac structure and function. Lagged associations between proteomic and echocardiographic measures were evaluated using the echocardiography measure obtained at the visit immediately subsequent to the biomarker. Linear mixed-effect models were adjusted for age, race (Black, White), cancer treatment (Dox, Tras, Dox + Tras), body mass index, smoking status, hypertension, diabetes, visit (baseline, 2–3 months, 12 months), and time interval between echocardiography acquisition and blood sample. P-values were corrected for multiple testing using the Benjamini and Hochberg procedure. Dashed lines indicate the significance level of FDR .05. Dots are colour-coded according to Olink panel. LVEF, left ventricular ejection fraction LV: left ventricular LA: left atrial. (*B*) Magnitude of the associations for proteins that were significant in the lagged analysis. Heat map of the magnitude of beta coefficients for significant proteins derived from the linear mixed-effect models. Red indicates positive associations, and blue indicates negative associations, with a darker colour indicating a larger absolute value. Poly(U)-specific endoribonuclease (ENDOU) had the strongest positive association with *E/e*′. Platelet glycoprotein V (GP5) had the strongest positive association with left atrial volume index. Enteropeptidase (TMPRSS15) and Prokineticin-1 (PROK1) had the strongest association with left ventricular mass index in terms of magnitude (negative and positive association, respectively). Ras GTPase-activating protein 1 (RASA1) and ubiquitin-associated domain-containing protein 1 (UBAC1) had the strongest association with longitudinal strain in terms of magnitude (negative and positive association, respectively). LAVi, left atrial volume index; LVMi: left ventricular mass index

Next, we determined the associations between the circulating proteome and echocardiographic measures at the subsequent visit in our lagged analyses. There were no proteins associated with LVEF; however, there were 113 proteins associated with longitudinal strain; two with LA volume index; two with *E*/*e*′; and three with LV mass index (Figure 3A and B, Supplementary data online, Table S3). Three proteins overlapped in the contemporaneous and lagged analyses and were associated with longitudinal strain and LA volume index (see Supplementary data online, Figure S3).

We further explored whether the associations between the above-identified proteomic markers and echocardiographic measures (a total of 210 protein-echocardiographic measure pairs in the contemporaneous and lagged analyses) differed according to cancer treatment regimen (Dox, Tras, or Dox + Tras). There was no evidence of effect modification by treatment in the association between protein markers and echocardiographic measures (see Supplementary data online, Tables S4–5). Similarly, we explored if there was effect modification by race, and there was no evidence for an interaction between race and proteome on the association with cardiac function and remodelling (see Supplementary data online, Tables S6–7).

### Pathway enrichment analysis

**Figure 4:**
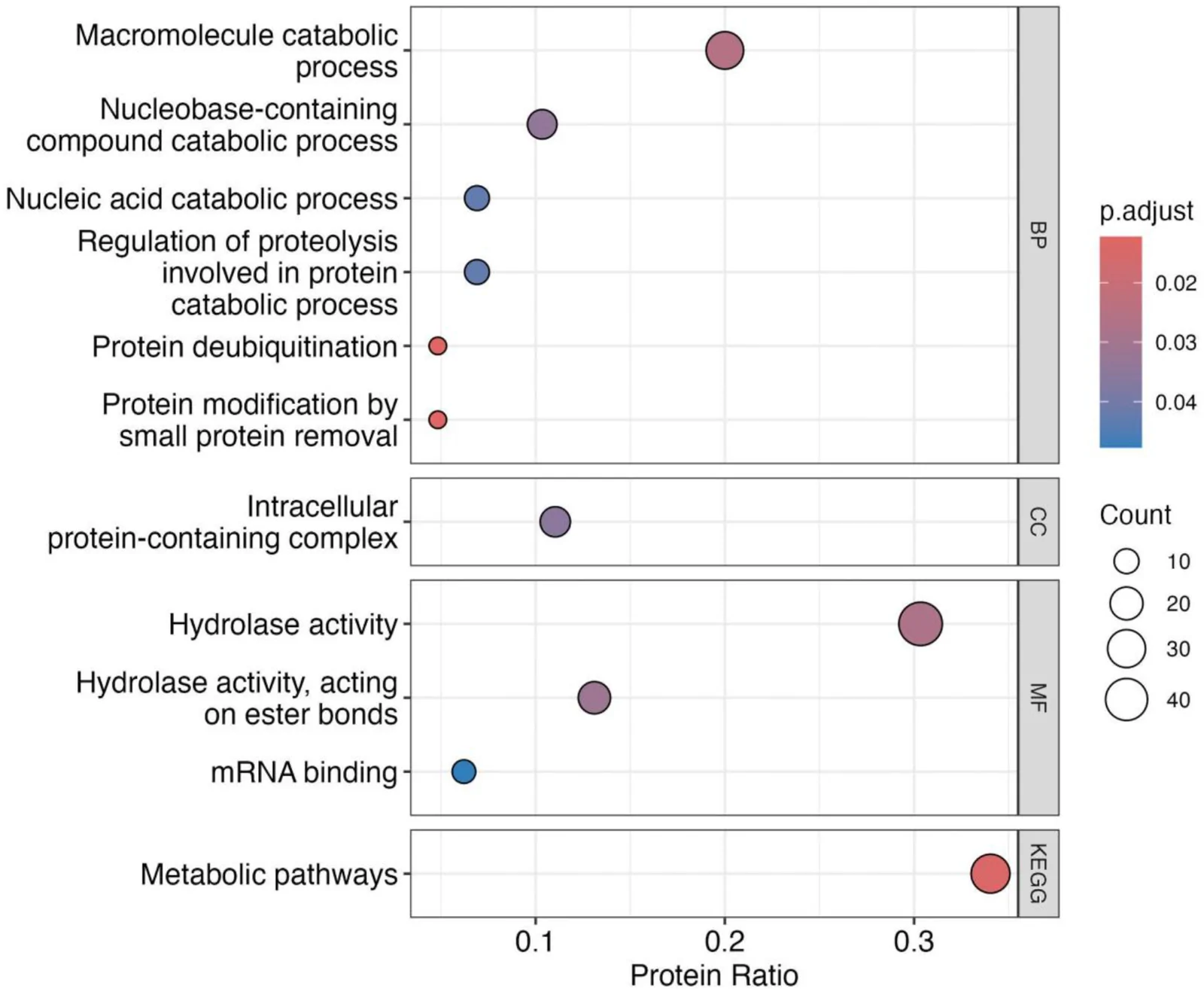
Pathway enrichment analysis of all proteins that were significantly associated with cardiac function. Proteins that exceeded the FDR significant threshold in the contemporaneous and lagged analyses for their association with echocardiographic measures for cardiac function (LVEF, longitudinal strain) served as a candidate set for the pathway enrichment analysis. The dotplot shows the significantly enriched Gene Ontology and KEGG pathways. The colour of the dot indicates the magnitude of the adjusted P-value. The size of the dot represents the number of candidate proteins that are enriched in the given annotation. Protein ratio: proportion of candidate proteins that map to the given annotation. BP, biological process; CC, cellular component; MF, molecular function; KEGG, Kyoto Encyclopedia of Genes and Genomes

Among the proteins identified in the contemporaneous or lagged analyses, 147 were positively associated with cardiac systolic function (LVEF or longitudinal strain), while eight showed negative associations. Proteins with positive associations were statistically enriched for biological processes including protein deubiquitination, protein modification by small protein removal, and macromolecule catabolic process (Figure 4, Supplementary data online, Table S8). We also explored KEGG pathway enrichment analysis and found a link to global metabolic pathways (hsa01100, FDR, *P* = .01). Several proteins including tumour necrosis factor receptor-associated factor 2 (TRAF2), eosinophil cationic protein (ECP; RNASE3), and fused in sarcoma (FUS) appeared in multiple enriched GO pathways. No pathway was statistically significantly enriched among proteins with negative associations.

### Associations between metabolomics and cardiac structure and function

**Figure 5:**
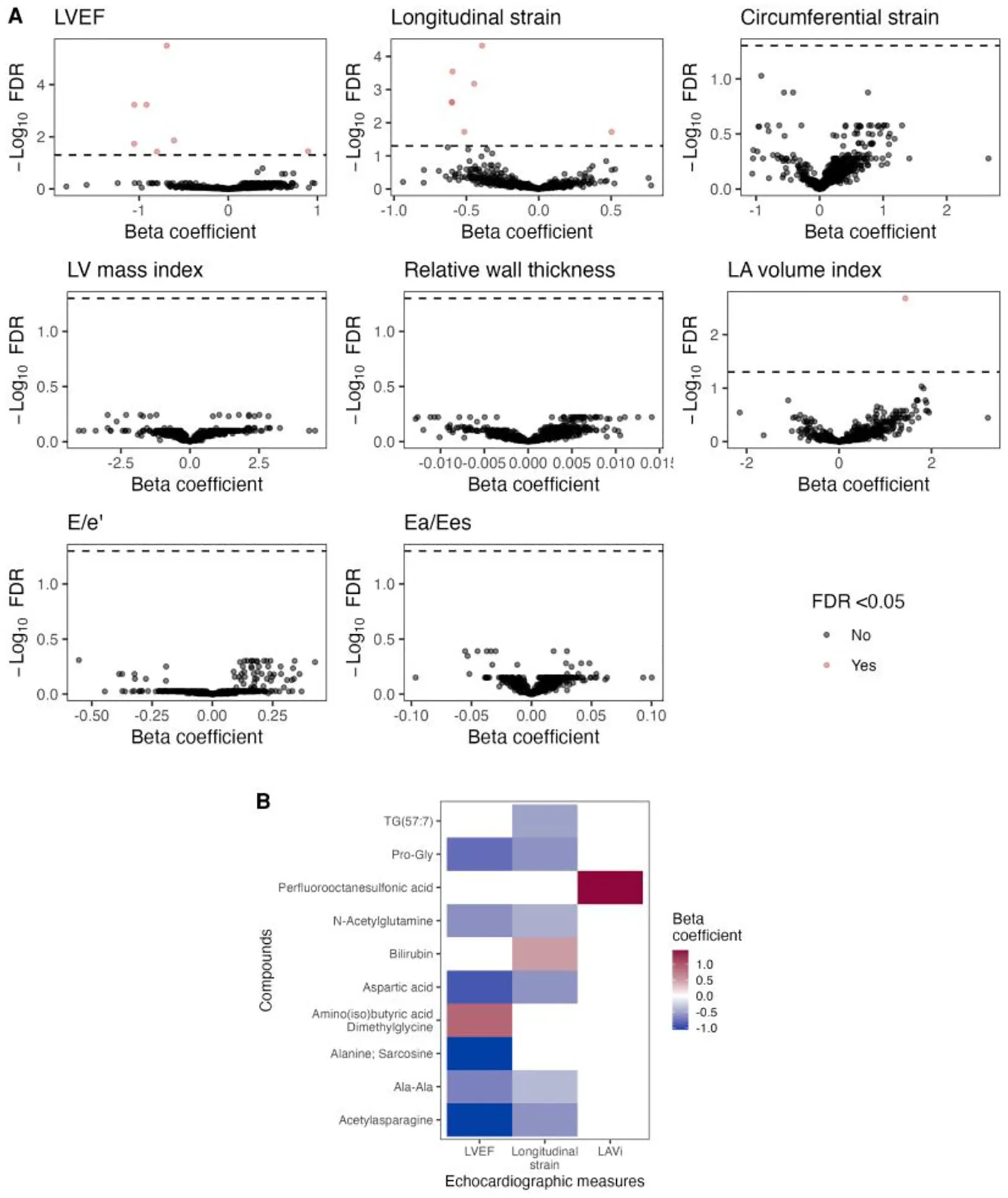
(*A*) Volcano plot for the contemporaneous associations between all metabolites and cardiac structure and function. Contemporaneous associations between metabolomic and echocardiographic measures were evaluated using measures obtained at the same visit. Linear mixed-effect models were adjusted for age, race (Black, White), cancer treatment (Dox, Tras, Dox + Tras), body mass index, smoking status, hypertension, diabetes, visit (baseline, 2–3 months, 12 months), and time interval between echocardiography acquisition and blood sample. *P*-values were corrected for multiple testing using the Benjamini and Hochberg procedure. Dashed lines indicate the significance level of FDR .05. Dots that passed the significance level are highlighted red. LVEF, left ventricular ejection fraction; LV, left ventricular; LA, left atrial. (*B*) Magnitude of the associations for metabolites that were significant in the contemporaneous analysis. Heat map of the magnitude of beta coefficients for significant metabolites derived from the linear mixed-effect models. Red indicates positive associations, and blue indicates negative associations, with a darker colour indicating a larger absolute value. LVEF, left ventricular ejection fraction; LAVi, left atrial volume index

Contemporaneous and lagged associations between the circulating metabolome and echocardiographic measures were also evaluated. Seven metabolites were associated with LVEF; seven with longitudinal strain; and one with LA volume index in contemporaneous analysis (Figure 5A and B and Supplementary data online, Table S9). Among these, five metabolites overlapped between LVEF and longitudinal strain, including *n*-acetylglutamine, aspartic acid, acetylasparagine, alanyl-alanine, and prolyl-glycine.

**Figure 6:**
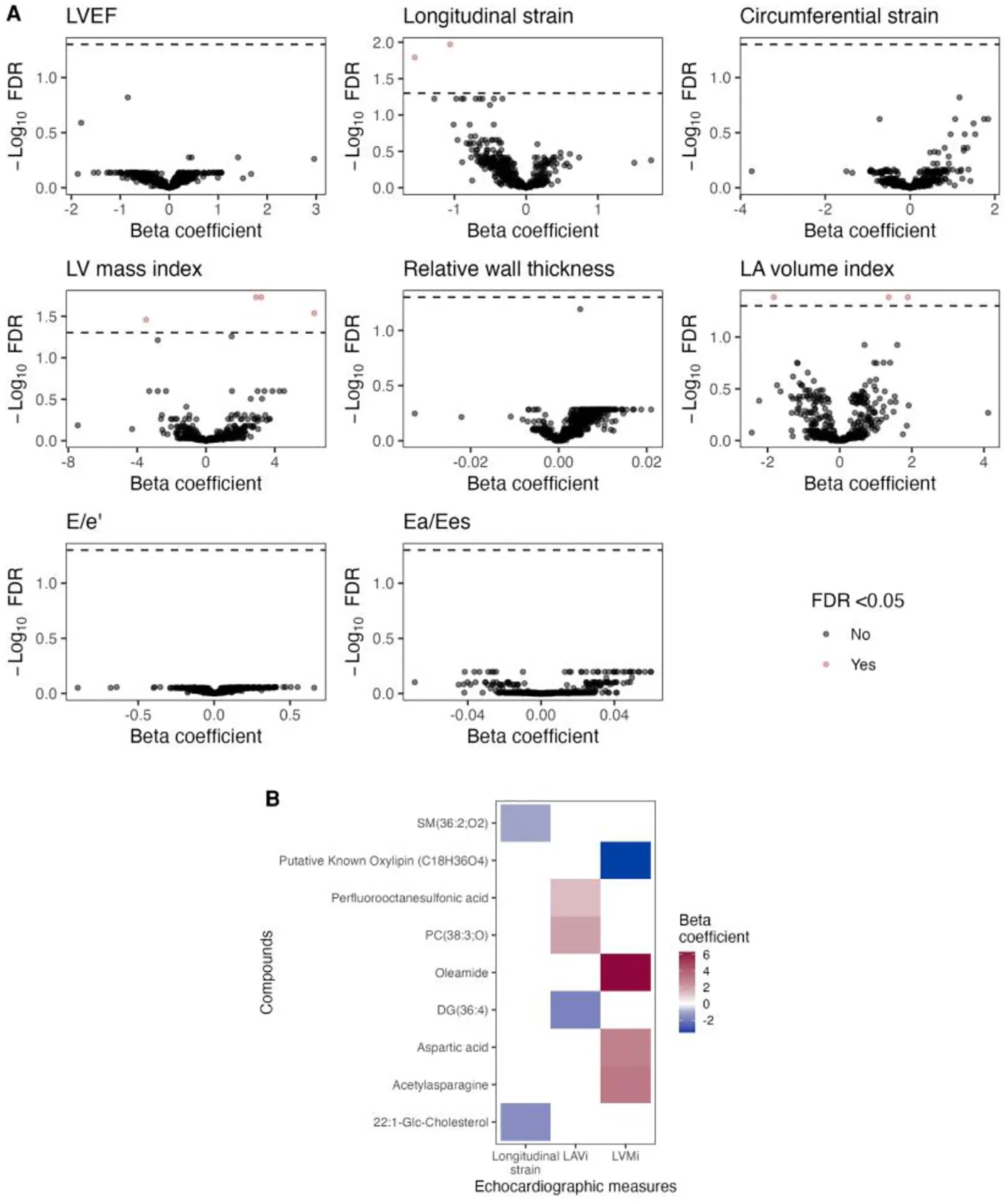
(*A*) Volcano plot for the lagged associations between all metabolites and cardiac structure and function. Lagged associations between metabolomic and echocardiographic measures were evaluated using the echocardiography measure obtained at the visit immediately subsequent to the biomarker. Linear mixed-effect models were adjusted for age, race (Black, White), cancer treatment (Dox, Tras, Dox + Tras), body mass index, smoking status, hypertension, diabetes, visit (baseline, 2–3 months, 12 months), and time interval between echocardiography acquisition and blood sample. *P*-values were corrected for multiple testing using the Benjamini and Hochberg procedure. Dashed lines indicate the significance level of FDR .05. Dots that passed the significance level are highlighted red. LVEF, left ventricular ejection fraction; LV, left ventricular; LA, left atrial. (*B*) Magnitude of the associations for metabolites that were significant in the lagged analysis. Heat map of the magnitude of beta coefficients for significant metabolites derived from the linear mixed-effect models. Red indicates positive associations, and blue indicates negative associations, with a darker colour indicating a larger absolute value. LAVi, left atrial volume index; LVMi, left ventricular mass index

In the lagged analysis, no metabolites were significantly associated with LVEF. However, two metabolites showed significant associations with longitudinal strain; four with LV mass index; and three with LA volume index (Figure 6A and B and Supplementary data online, Table S10). The overlap in metabolites across echocardiographic measures in contemporaneous and lagged analyses is summarized in Supplementary data online, Figure S4. These three overlapping metabolites included perfluorooctanesulfonic acid (PFOS), aspartic acid, and acetylasparagine.

**Figure 7:**
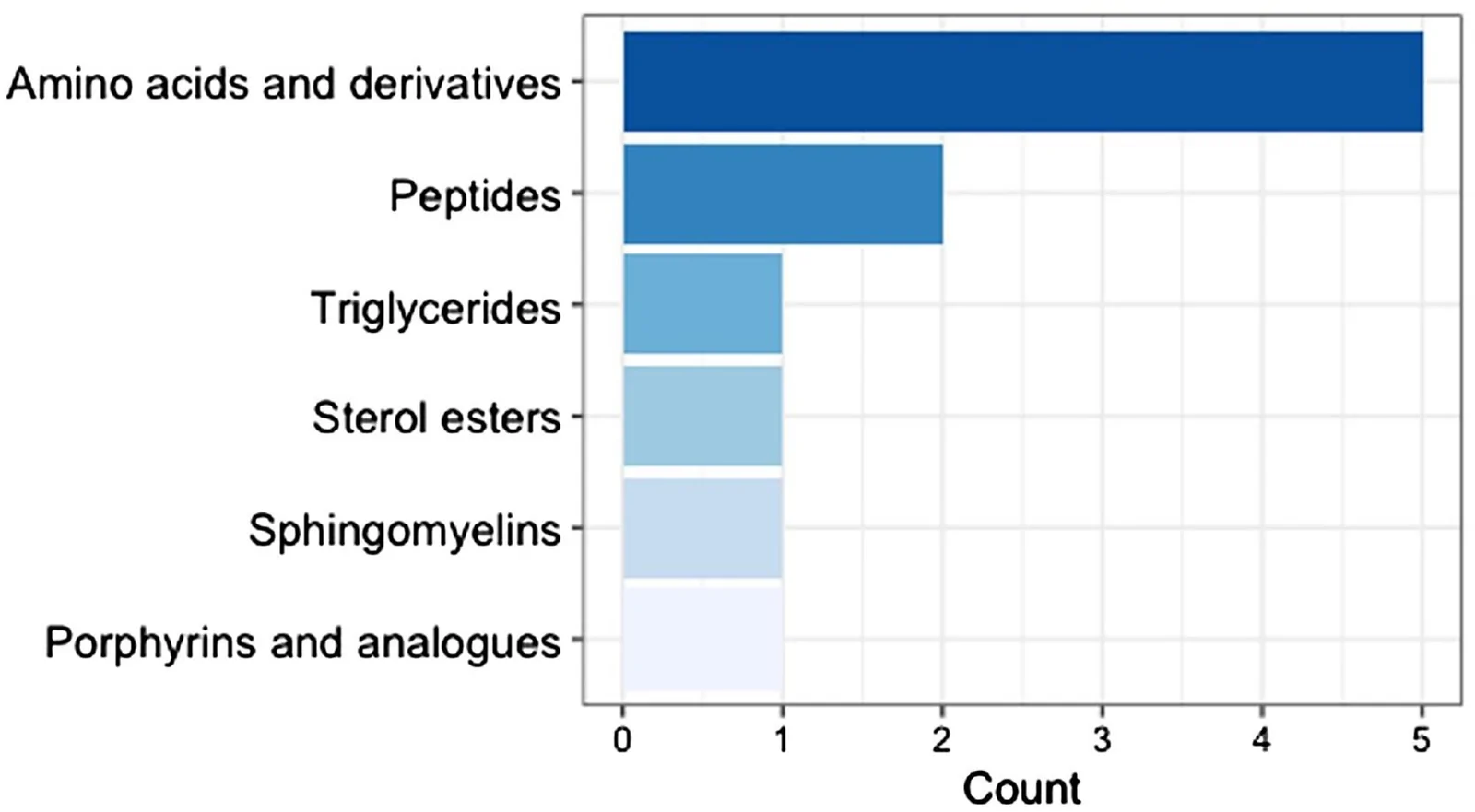
Biochemistry class of all metabolites that were significantly associated with cardiac function. Barplot showing the number of metabolites classified under each biochemistry class

Of the 11 metabolites that demonstrated significant associations with cardiac systolic function (LVEF or longitudinal strain) in the contemporaneous and lagged analyses, most belonged to amino acids and derivatives, followed by peptides (Figure 7).

### Proteomic/metabolomic biomarker correlations

Protein-metabolite correlations for measures of cardiac systolic function (LVEF or longitudinal strain) are illustrated in Supplementary data online, Figure S5. The absolute value of the strongest correlations was on the order of .28–.41 across all timepoints. At the 2–3 month timepoint, bilirubin, which demonstrated an association with longitudinal strain, had one of the most negative correlations with aspartic protease DNA damage inducible 1 homolog 2 (DDI2, *ρ* = −.4). DDI2 was associated with longitudinal strain and appeared in our proteomic-enriched pathway analysis, particularly in the macromolecule catabolic process pathway.

## Discussion

In this comprehensive analysis aimed at determining the longitudinal associations between the circulating proteome and metabolome and echocardiographic measures of structure and function in breast cancer patients receiving cardiotoxic therapy, we report five main findings. First, there are significant associations between circulating proteomic and metabolomic markers and echocardiographic measures in breast cancer patients receiving cardiotoxic cancer therapy. Second, notable protein markers significantly associated with cardiac function include CTSC and S100A13. Third, the circulating proteomic markers associated with cardiac function were mainly linked to biological processes, including protein deubiquitination, protein modification by small protein removal, macromolecule catabolic process, and global metabolic pathways. Fourth, metabolites significantly associated with measures of cardiac function included: *n*-acetylglutamine, aspartic acid, acetylasparagine, alanyl-alanine, and prolyl-glycine. Fifth, metabolites that demonstrated significant associations with cardiac function primarily belonged to amino acids and derivatives, followed by peptides. While we acknowledge the important need for further validation of our findings and a deeper understanding of the biology of these markers, there are several key strengths of our study. These include: conceptual innovation in our study design and overarching hypotheses with the incorporation of detailed longitudinal measures and evaluation of both contemporaneous (‘diagnostic’) and lagged (‘predictive’) associations over extended follow-up; technical innovation in our state-of-the-art -omics evaluation and core-lab quantified measures; methodologic innovation with rigor in our study design and statistical analysis with the blinded, centralized quantitation of both -omics and echocardiography measures, use of multiple testing, and incorporation of the largest sample size to date; generalizability with the study of a modern day breast cancer cohort treated with contemporary regimens.

Only a few human studies have applied omics approaches to investigate potential biomarkers for cancer therapy-related cardiotoxicity. We acknowledge that these findings have been variable across these studies, and we hypothesize that this is related to the small sample sizes of these prior studies, the heterogeneity of these cohorts in terms of population characteristics, including cancer type, treatment regimen, and time since cancer therapy exposure, and the exact question of interest. For example, Poudel *et al*. identified 27 proteins that discriminated between 75 asymptomatic survivors with late cardiomyopathy (~20 years from cancer diagnosis) and 75 without.^13^ Another study of 95 patients with diffuse large B-cell lymphoma planned to receive doxorubicin-based chemotherapy (during a median follow-up of 69 months), found interleukin-1 receptor type 1 to be correlated with cardiovascular disease.^12^ Another -omics study of 136 women with breast cancer who received sequential anthracycline and trastuzumab therapy focused on miRNAs, cytokines, and chemokines identified primarily vascular and inflammatory biomarkers.^32^

Among the proteomic targets that showed significant associations with cardiac structure and function, CTSC is worth highlighting, although we fully acknowledge the need for external validation of our findings to further define its potential for predictive accuracy, as well as a need to understand the biologic mechanisms and specificity of CTSC to cancer treatment and cancer itself, and how this protein relates to cardiac function and remodelling in non-cancer populations. CTSC (also known as dipeptidyl peptidase I) is a lysosomal cysteine protease involved in the activation of granule serine proteases in immune cells, particularly neutrophils. In human vascular tissue, CTSC gene and protein expression were increased in ruptured compared with stable carotid atherosclerotic plaques and localized to macrophage- and foam cell–rich regions.^33^ Hematopoietic CTSC deficiency in low-density lipoprotein receptor–deficient mice was associated with reduced atherosclerotic plaque burden across multiple vascular beds, linking leucocyte CTSC to atherosclerosis severity.^33^ In human coronary artery tissue, a small proteomics study linked CTSC expression to inflammatory signalling pathways.^34^

CTSC has also been examined in acute inflammatory injury models relevant to cardiovascular disease. In a rat heart transplantation ischaemia–reperfusion model, pharmacologic CTSC inhibition was associated with reduced neutrophil serine protease activity, lower inflammatory and oxidative injury markers, and improved graft functional parameters after reperfusion.^35^ Separately, CTSC inhibition has been shown to suppress neutrophil serine protease activity and reduce neutrophil extracellular trap formation in experimental models, with associated improvement in inflammatory vasculitis.^36^ In vascular smooth muscle cells, cathepsin C–dependent activation of chymase has been shown to mediate non-canonical processing of interleukin-1β, with evidence of chymase expression in the fibrous cap of human carotid plaques.^37^ Collectively, these studies support associations between CTSC and cardiovascular disease, particularly in settings characterized by immune activation, vascular inflammation, and ischaemia–reperfusion injury.

Members of the cysteine protease cathepsin family have been associated with heart failure severity and prognosis, including large prospective cohort data demonstrating associations between circulating cathepsin D levels and clinical outcomes in patients with chronic heart failure.^38^ Experimental and translational studies have further implicated cysteine cathepsins in inflammatory and extracellular matrix–related pathways relevant to cardiovascular and myocardial remodelling.^39^

In our previous work, we found that the cathepsin S subtype was significantly different in breast cancer patients who experienced doxorubicin and trastuzumab cancer therapy-related cardiac dysfunction, compared to matched controls.^40^ Altogether, cathepsin proteases show promise as biomarkers and potential therapeutic targets in cardiovascular disease and specifically cardio-oncology, although we acknowledge the need for additional validation and mechanistic data to further clarify the function of particular subtypes.

Moreover, S100A13 had some of the strongest negative associations with longitudinal strain. S100A13 is involved in calcium homeostasis and serves as a cargo protein to facilitate non-classical export of fibroblast growth factor-1 and interleukin 1-α in response to cellular stress, which in turn promotes angiogenesis, inflammation, and cellular senescence.^41^ In a previous Mendelian randomization study, S100A13 was associated with a higher risk of atherosclerotic cardiovascular disease.^42^ In a study of nearly 49 000 UK Biobank participants, unsupervised geometric deep learning was applied to 3D cardiac MRI-derived LV meshes to extract latent phenotypes of LV morphology, and genome-wide association analyses identified S100A13 as a suggestive locus, highlighting a potential role in influencing cardiac structure.^43^ Other proteins in the S100 family, such as S100A8/9, contribute to both cancer and cardiovascular disease.^44^ In our literature review of the significant proteins most strongly associated with each echocardiographic measure, we found several additional markers with plausible roles in cardiovascular disease (see Supplementary data online, Table S1).

In our pathway analysis, proteins indicative of protein deubiquitination, protein modification by small protein removal, and macromolecule catabolic process were significant. In particular, TRAF2, which was associated with longitudinal strain, is an E3 ubiquitin-protein ligase that regulates proteostasis and apoptosis. TRAF2 has cytoprotective effects in the mouse heart mediated by crosstalk between the canonical and non-canonical NFkB signalling pathways, and overexpression of TRAF2 mitigates doxorubicin-induced cardiac dysfunction in mice, and inhibition is associated with increased cardiomyocyte death.^45,46^ ECP, encoded by *RNASE3*, is an inflammatory marker with known cytotoxic properties, and was also associated with longitudinal strain. A Mendelian randomization study showed that higher genetically predicted levels of ECP were associated with an increased risk of gestational hypertension, and that ECP promoted atherosclerosis and vascular calcification in mice.^47,48^ FUS was associated with LVEF. FUS is a DNA and RNA-binding protein involved in transcriptional regulation and the DNA repair response, and has been associated with increased fibrosis and reduced angiogenesis in murine models of myocardial infarction.^49,50^ Collectively, these proteomic markers offer potential biological insights into cancer therapy-induced cardiotoxicity and, pending additional basic investigations, may have a potential role in the diagnosis, risk prediction, and development of targeted cardioprotective therapies.

There is limited evidence of metabolomic disturbances related to cancer therapy-related cardiotoxicity in human studies, with only a small number of human studies of lesser sample size that have explored metabolomics alterations in cancer patients who received potentially cardiotoxic therapies. For example, using targeted mass spectrometry-based metabolomics, Asnani *et al*. observed different levels of citric acid and aconitic acid in 38 women with breast cancer treated with anthracyclines and trastuzumab, comparing those who developed cardiotoxicity to those who did not.^15^ In another targeted metabolomics study assessing systemic oxidative stress, Thonusin *et al*. found that changes in selected fatty acid-derived acylcarnitines 2 weeks after completion of doxorubicin treatment in 74 breast cancer patients were correlated with LVEF.^16^ Lal *et al*. reported that metabolites relevant to inflammation and cancer progression were significantly associated with LV dysfunction in 50 cancer patients.^14^ Animal studies of doxorubicin-induced cardiotoxicity have identified perturbations in amino acid metabolism, including pathways involving alanine, aspartate, and glutamate; however, no other human studies have corroborated this.^16,51^ As it relates to measures of LV mass, an animal study by Ritterhoff *et al*. demonstrated that aspartate accumulation is a requirement for cardiomyocyte hypertrophy, driven by a pathological substrate switch and hypertrophic stimuli.^52^ Importantly, our study is among the first to suggest the association between aspartic acid and cancer-therapy related cardiac remodelling in a human cohort. Three metabolites: (i) aspartic acid, an intermediate in the malate-aspartate shuttle; (ii) acetylasparagine were each associated with contemporaneous LVEF and longitudinal strain and lagged LV mass index; and (iii) *n*-acetylglutamine, implicated in amino acid catabolism, acetyl-CoA availability, and nitrogen handling, was associated with contemporaneous LVEF and longitudinal strain. These metabolites, involved in energy metabolism, mitochondrial function, nitrogen handling, and protein turnover, provide a potential biological link to cancer therapy-related cardiac remodelling and dysfunction. Our metabolomics platform included acylcarnitines (with coverage of long-, middle-, and short-chain species) and intermediates of the tricarboxylic acid cycle and fatty-acid oxidation. However, these did not emerge as statistically significant associations with cardiac function or remodelling.

Additionally, in our metabolic profiling, an exogenous environmental contaminant PFOS, was associated with LA volume index in both contemporaneous and lagged models. PFOS is an anthropogenic fluorosurfactant widely used in consumer and industrial products, and is considered a ‘forever chemical’ due to its resistance to natural degradation, and has been associated with diverse adverse health outcomes.^53^ Epidemiologic studies have found that exposure to PFOS was associated with the development of cardiovascular disease as well as excess mortality from both cardiovascular disease and cancer.^54–58^

Our study provides important innovation, new translational knowledge in biomarker discovery, and further insights into biological processes important in cancer therapy-related cardiotoxicity. Prior to clinical implementation of any of these markers, however, there is an important need for additional study with external validation. Implementation into routine clinical practice will also likely entail a parsimonious panel of multiple markers with the strongest and most reproducible associations. However, our work lays the necessary foundation for these next steps. Nonetheless, the limitations of this study should be acknowledged. First, although one of the largest reports to date in cardio-oncology, we still had limited power to assess associations with clinical outcomes, as well as to perform additional subgroup analyses. Analyses regarding the risk of incident cancer therapy-related cardiac dysfunction should be interpreted as exploratory and supportive, given that the same cohort was used for discovery, and both proteomics and metabolomics were quantified on a relative, and not absolute, scale. Although we further emphasize the critical need for external validation of these findings, we note that the cathepsin family of proteins was also significant in our prior work.^40^ Still, we acknowledge that the associations with CTSC, in particular, carry a risk of overfitting, and there is a need for a deeper mechanistic understanding of the biology of CTSC as it relates to cancer, cancer treatment, and cardiovascular disease, specifically cardiomyopathy and heart failure in the general population. Third, given the observational nature of this study, causality between the circulating proteome, metabolome, and cardiac function and remodelling needs to be further demonstrated in basic mechanistic investigations. Fourth, we did not have as a comparator cohort patients with cancer not exposed to cardiotoxic therapy or a cohort of non-cancer patients with heart failure. However, we also note several strengths of our study, including the detailed longitudinal phenotyping with comprehensive clinical, biomarker, and echocardiographic data, as well as rigorous core lab analysis of proteomic, metabolomic, and imaging data, and comprehensive statistical analysis. Importantly, our study provides new discovery-level insights and potentially novel biomarkers for the diagnosis and prediction of cancer therapy-related cardiac dysfunction.

In conclusion, in this prospective longitudinal cohort of breast cancer patients receiving cardiotoxic therapies, we identified several novel associations between the circulating proteome and metabolome and cardiac structure and function. Our findings provide important insights into the role of key pathways in cancer therapy-related cardiac dysfunction and remodelling.

## Supplementary Material

Supplementary Material

Supplementary data are available at European Heart Journal online.

## Figures and Tables

**Table 1:** Demographics and clinical characteristics of study participants

Characteristic	CCT cohort (*n* = 547)
Age at enrolment, years	50 (41, 58)
Race	
Black	144 (26.3%)
White	403 (73.7%)
AJCC stages at diagnosis	
Stage 1	123 (22.5%)
Stage 2	291 (53.2%)
Stage 3	124 (22.7%)
Stage 4	9 (1.6%)
Laterality	
Bilateral	23 (4.3%)
Left	258 (48.0%)
Right	256 (47.7%)
Cancer therapy regimen	
Doxorubicin (Dox)	332 (60.7%)
Doxorubicin + Trastuzumab (Dox + Tras)	79 (14.4%)
Trastuzumab (Tras)	136 (24.9%)
Radiotherapy	
None	162 (29.7%)
Left-sided	191 (35.0%)
Right-sided	175 (32.1%)
Bilateral	18 (3.3%)
Tobacco use	
Current	34 (6.2%)
Former	182 (33.3%)
Never	331 (60.5%)
Body mass index, kg/m^2^	27 (23, 32)
Systolic blood pressure, mmHg	126 (115, 137)
Diastolic blood pressure, mmHg	76 (70, 82)
Heart rate, beats per minute	78 (70, 88)
History of hypertension	170 (31.1%)
History of diabetes	49 (9.0%)
History of hyperlipidaemia	137 (25.2%)
History of cardiovascular disease	55 (10.2%)
Cardiovascular medications	221 (40.7%)
Beta blockers	52 (9.5%)
ACEis or ARBs	85 (15.5%)
Calcium-channel blockers	50 (9.1%)
Diuretics	74 (13.5%)
Statins	78 (14.4%)

Data are presented as median (Quartile 1, Quartile 3) or *n* (%).

History of cardiovascular disease defined by history of coronary disease, arrhythmia, heart failure, stroke, or cardiac surgery.

ACEis, angiotensin-converting enzyme inhibitors; ARBs, angiotensin receptor blockers.

## Data Availability

Data sharing is not available for this study.

## Translational Perspective

Proteomics and metabolomics profiling provide insights into the underlying biological perturbations that occur in breast cancer patients receiving cardiotoxic therapies. The analyses revealed longitudinal changes in circulating proteomic and metabolomic markers and their associations with cardiac function and remodelling. These findings provide new knowledge in potential biomarkers and pathways in cardio-oncology, which hold promise for identifying patients at risk for cancer therapy-related cardiac dysfunction and adverse remodelling. There is an important need for further validation of these findings and a deeper understanding of the biology of these biomarkers to advance our translational perspective.
