# Supplementary material for: Cardiac remodelling and dysfunction in cancer patients receiving cardiotoxic therapies: proteomic and metabolomic profiling
Source: Eur Heart J. Author manuscript; Available in PMC 2026 Jul 13. (PMC13359040; doi:10.1093/eurheartj/ehag487)
Supplement: Supplementary Material [file NIHMS2193983-supplement-Supplementary_Material.docx]

**Supplemental Material**

**Supplemental Methods**

**Metabolomics**

***Sample analysis***

Samples were analyzed by rapid liquid chromatography-mass spectrometry (rLC-MS). rLC-MS utilizes rapid nano-valve switching in tandem with a three-pump system delivering a multi-tiered isocratic elution coupled to a high-resolution Bruker TIMS TOF Pro 2 mass spectrometer (Bruker Corporation, Billerica, MA, USA). Samples were injected onto a custom packed silica-based mixed mode column that allows for both reverse phase and weak ion pairing retention mechanisms. Data acquisition occurred in both positive and negative electrospray ionization modes with an injection-to-injection cycle time of 52 seconds. Samples were eluted using mobile phases starting with (20:80) Methanol: Water with 0.05% acetic acid and 2.5 mM ammonium acetate for positive ion mode or (35:65) Methanol: Water with 0.02% acetic acid and 5 mM ammonium acetate for negative ion mode and ending with (80:20) Methanol: IPA with 0.1% acetic acid and 7.5 mM ammonium acetate for positive ion mode or (70:30) Methanol: IPA with 0.1% acetic acid and 20 mM ammonium acetate for negative ion mode.

***Quality control***

For each 384-well plate run, the QC was the first preparation step to monitor matrix effects; bulk pre-aliquoted commercial pooled plasma (BioIVT) was placed in wells A1, D12, and H12 of each 96-well patient plasma plate and prepared identically to samples (internal bracket QC sample); bulk pre-aliquoted commercial pooled plasma was prepared external to the 96-well plate by hand (external bracket QC sample), and a preparation blank was prepared during each 384-well plate to assess background.

**Supplemental Table 1** Individual proteins associated with cardiac structure and function in the contemporaneous analysis

| **Echocardiographic measure** | **Gene** | **Protein name** | **Panel** | **Beta** | **P value** | **FDR** |
| --- | --- | --- | --- | --- | --- | --- |
| LA volume index | AMBN | Ameloblastin | Inflammation | -2.817662 | 0.000056 | 0.034926 |
| LA volume index | CTSC | Cathepsin C / Dipeptidyl peptidase 1 | Inflammation | -1.695483 | 0.000052 | 0.034926 |
| LA volume index | AHNAK | Neuroblast differentiation-associated protein AHNAK | Cardiometabolic II | -1.255049 | 0.000506 | 0.048107 |
| LA volume index | HGF | Hepatocyte growth factor | Inflammation | -1.050395 | 0.000667 | 0.048107 |
| LA volume index | IL1RN | Interleukin-1 receptor antagonist protein | Inflammation | -0.980385 | 0.000370 | 0.048107 |
| LA volume index | SAFB2 | Scaffold attachment factor B2 | Neurology II | -0.941014 | 0.000151 | 0.037756 |
| LA volume index | PAXX | Protein PAXX | Inflammation II | -0.938607 | 0.000379 | 0.048107 |
| LA volume index | KCTD5 | BTB/POZ domain-containing protein KCTD5 | Neurology II | -0.907147 | 0.000606 | 0.048107 |
| LA volume index | NAGK | N-acetyl-D-glucosamine kinase | Cardiometabolic II | -0.879521 | 0.000236 | 0.045421 |
| LA volume index | GIMAP8 | GTPase IMAP family member 8 | Oncology II | -0.832133 | 0.000203 | 0.041940 |
| LA volume index | MPO | Myeloperoxidase | Neurology | -0.778579 | 0.000410 | 0.048107 |
| LA volume index | RPE | Ribulose-phosphate 3-epimerase | Oncology II | -0.765469 | 0.000499 | 0.048107 |
| LA volume index | RALY | RNA-binding protein Raly | Oncology II | -0.734393 | 0.000078 | 0.034926 |
| LA volume index | ILKAP | Integrin-linked kinase-associated serine/threonine phosphatase 2C | Neurology | -0.732671 | 0.000170 | 0.037756 |
| LA volume index | BAG4 | BAG family molecular chaperone regulator 4 | Inflammation II | -0.725979 | 0.000796 | 0.048925 |
| LA volume index | ERI1 | 3'-5' exoribonuclease 1 | Oncology II | -0.719186 | 0.000562 | 0.048107 |
| LA volume index | KLF4 | Krueppel-like factor 4 | Neurology II | -0.715481 | 0.000163 | 0.037756 |
| LA volume index | CWC15 | Spliceosome-associated protein CWC15 homolog | Oncology II | -0.711330 | 0.000274 | 0.048107 |
| LA volume index | FOXJ3 | Forkhead box protein J3 | Inflammation II | -0.696510 | 0.000506 | 0.048107 |
| LA volume index | PQBP1 | Polyglutamine-binding protein 1 | Oncology | -0.682931 | 0.000721 | 0.048107 |
| LA volume index | WDR46 | WD repeat-containing protein 46 | Oncology II | -0.670451 | 0.000435 | 0.048107 |
| LA volume index | NADK | NAD kinase | Cardiometabolic | -0.666276 | 0.000528 | 0.048107 |
| LA volume index | THAP12 | 52 kDa repressor of the inhibitor of the protein kinase | Oncology II | -0.665774 | 0.000613 | 0.048107 |
| LA volume index | CASP10 | Caspase-10 | Neurology | -0.650846 | 0.000116 | 0.034926 |
| LA volume index | TDP1 | Tyrosyl-DNA phosphodiesterase 1 | Oncology II | -0.640039 | 0.000552 | 0.048107 |
| LA volume index | MRI1 | Methylthioribose-1-phosphate isomerase | Neurology II | -0.631255 | 0.000476 | 0.048107 |
| LA volume index | ELOA | Elongin-A | Oncology | -0.601260 | 0.000087 | 0.034926 |
| LA volume index | SRP14 | Signal recognition particle 14 kDa protein | Oncology | -0.597609 | 0.000121 | 0.034926 |
| LA volume index | SNRPB2 | U2 small nuclear ribonucleoprotein B'' | Neurology II | -0.594191 | 0.000333 | 0.048107 |
| LA volume index | HDGFL2 | Hepatoma-derived growth factor-related protein 2 | Neurology II | -0.581225 | 0.000720 | 0.048107 |
| LA volume index | CPPED1 | Serine/threonine-protein phosphatase CPPED1 | Neurology | -0.580749 | 0.000715 | 0.048107 |
| LA volume index | BCL2L15 | Bcl-2-like protein 15 | Inflammation II | -0.571703 | 0.000738 | 0.048107 |
| LA volume index | FGR | Tyrosine-protein kinase Fgr | Neurology | -0.558355 | 0.000019 | 0.034926 |
| LA volume index | NPM1 | Nucleophosmin | Neurology | -0.557307 | 0.000115 | 0.034926 |
| LA volume index | APEX1 | DNA-(apurinic or apyrimidinic site) endonuclease | Oncology | -0.551094 | 0.000056 | 0.034926 |
| LA volume index | MKI67 | Proliferation marker protein Ki-67 | Inflammation II | -0.540147 | 0.000396 | 0.048107 |
| LA volume index | DUT | Deoxyuridine 5'-triphosphate nucleotidohydrolase, mitochondrial | Oncology II | -0.535460 | 0.000589 | 0.048107 |
| LA volume index | BAP18 | Chromatin complexes subunit BAP18 | Neurology II | -0.534140 | 0.000322 | 0.048107 |
| LA volume index | TOP2B | DNA topoisomerase 2-beta | Inflammation II | -0.532275 | 0.000750 | 0.048107 |
| LA volume index | SMNDC1 | Survival of motor neuron-related-splicing factor 30 | Oncology II | -0.528381 | 0.000090 | 0.034926 |
| LA volume index | TOR1AIP1 | Torsin-1A-interacting protein 1 | Cardiometabolic II | -0.495920 | 0.000788 | 0.048925 |
| LA volume index | AZU1 | Azurocidin | Cardiometabolic | -0.471930 | 0.000695 | 0.048107 |
| LA volume index | RAB44 | Ras-related protein Rab-44 | Oncology II | -0.456910 | 0.000648 | 0.048107 |
| LA volume index | PADI4 | Protein-arginine deiminase type-4 | Neurology | -0.415864 | 0.000651 | 0.048107 |
| LA volume index | NCF2 | Neutrophil cytosol factor 2 | Inflammation | -0.400187 | 0.000370 | 0.048107 |
| LA volume index | MNDA | Myeloid cell nuclear differentiation antigen | Cardiometabolic | -0.376035 | 0.000317 | 0.048107 |
| LA volume index | GPD1 | Glycerol-3-phosphate dehydrogenase [NAD(+)], cytoplasmic | Oncology II | 0.859321 | 0.000536 | 0.048107 |
| LVEF | CASP1 | Caspase-1 | Neurology | 0.316252 | 0.000435 | 0.045665 |
| LVEF | FMNL1 | Formin-like protein 1 | Neurology | 0.320271 | 0.000173 | 0.043638 |
| LVEF | SKAP1 | Src kinase-associated phosphoprotein 1 | Neurology | 0.322196 | 0.000288 | 0.045665 |
| LVEF | DDX58 | Antiviral innate immune response receptor RIG-I | Oncology | 0.380201 | 0.000227 | 0.043638 |
| LVEF | IRAG2 | Inositol 1,4,5-triphosphate receptor associated 2 | Cardiometabolic | 0.389006 | 0.000569 | 0.045665 |
| LVEF | ILKAP | Integrin-linked kinase-associated serine/threonine phosphatase 2C | Neurology | 0.430300 | 0.000673 | 0.048596 |
| LVEF | VPS4B | Vacuolar protein sorting-associated protein 4B | Oncology II | 0.446383 | 0.000302 | 0.045665 |
| LVEF | SDC4 | Syndecan-4 | Cardiometabolic | 0.472065 | 0.000377 | 0.045665 |
| LVEF | GIMAP8 | GTPase IMAP family member 8 | Oncology II | 0.499336 | 0.000564 | 0.045665 |
| LVEF | MORC3 | MORC family CW-type zinc finger protein 3 | Neurology II | 0.510182 | 0.000639 | 0.048589 |
| LVEF | STX4 | Syntaxin-4 | Oncology | 0.521637 | 0.000673 | 0.048596 |
| LVEF | NMT1 | Glycylpeptide N-tetradecanoyltransferase 1 | Neurology II | 0.540424 | 0.000521 | 0.045665 |
| LVEF | ZHX2 | Zinc fingers and homeoboxes protein 2 | Neurology II | 0.550654 | 0.000522 | 0.045665 |
| LVEF | CHEK2 | Serine/threonine-protein kinase Chk2 | Cardiometabolic | 0.552167 | 0.000213 | 0.043638 |
| LVEF | RNASEH2A | Ribonuclease H2 subunit A | Neurology II | 0.562598 | 0.000555 | 0.045665 |
| LVEF | ARHGAP25 | Rho GTPase-activating protein 25 | Oncology | 0.598878 | 0.000562 | 0.045665 |
| LVEF | SH3BP1 | SH3 domain-binding protein 1 | Oncology II | 0.600721 | 0.000166 | 0.043638 |
| LVEF | FUS | RNA-binding protein FUS | Oncology | 0.637971 | 0.000432 | 0.045665 |
| LVEF | C7orf50 | Uncharacterized protein C7orf50 | Neurology II | 0.657566 | 0.000487 | 0.045665 |
| LVEF | ARSB | Arylsulfatase B | Oncology | 0.691102 | 0.000121 | 0.043638 |
| LVEF | TNFSF14 | Tumor necrosis factor ligand superfamily member 14 | Neurology | 0.691835 | 0.000327 | 0.045665 |
| LVEF | RCC1 | Regulator of chromosome condensation | Oncology II | 0.710050 | 0.000473 | 0.045665 |
| LVEF | EDAR | Tumor necrosis factor receptor superfamily member EDAR | Inflammation | 0.714257 | 0.000540 | 0.045665 |
| LVEF | HK2 | Hexokinase-2 | Cardiometabolic | 0.721042 | 0.000454 | 0.045665 |
| LVEF | HBEGF | Proheparin-binding EGF-like growth factor | Oncology | 0.723646 | 0.000114 | 0.043638 |
| LVEF | SUSD1 | Sushi domain-containing protein 1 | Cardiometabolic | 0.726355 | 0.000076 | 0.043612 |
| LVEF | ARID4B | AT-rich interactive domain-containing protein 4B | Neurology | 0.727257 | 0.000026 | 0.029900 |
| LVEF | GIMAP7 | GTPase IMAP family member 7 | Inflammation II | 0.730059 | 0.000478 | 0.045665 |
| LVEF | CEP164 | Centrosomal protein of 164 kDa | Inflammation | 0.762024 | 0.000217 | 0.043638 |
| LVEF | INPP1 | Inositol polyphosphate 1-phosphatase | Oncology | 0.766565 | 0.000179 | 0.043638 |
| LVEF | CD63 | CD63 antigen | Neurology | 0.775245 | 0.000595 | 0.046465 |
| LVEF | BTC | Probetacellulin | Oncology | 0.775446 | 0.000041 | 0.029900 |
| LVEF | DDX39A | ATP-dependent RNA helicase DDX39A | Inflammation II | 0.862328 | 0.000417 | 0.045665 |
| LVEF | IMPA1 | Inositol monophosphatase 1 | Neurology | 0.870707 | 0.000031 | 0.029900 |
| LVEF | GPC5 | Glypican-5 | Neurology | 1.025417 | 0.000535 | 0.045665 |
| LVEF | PLSCR3 | Phospholipid scramblase 3 | Neurology II | 1.094723 | 0.000393 | 0.045665 |
| LVEF | GLB1 | Beta-galactosidase | Neurology | 1.126202 | 0.000131 | 0.043638 |
| LVEF | CTSC | Cathepsin C / Dipeptidyl peptidase 1 | Inflammation | 1.197820 | 0.000006 | 0.017267 |
| LVEF | ANXA11 | Annexin A11 | Inflammation | 1.295231 | 0.000541 | 0.045665 |
| LVEF | SSH3 | Protein phosphatase Slingshot homolog 3 | Oncology II | 1.783807 | 0.000224 | 0.043638 |
| Longitudinal strain | KLK13 | Kallikrein-13 | Oncology | -0.678505 | 0.000033 | 0.046098 |
| Longitudinal strain | S100A13 | Protein S100-A13 | Inflammation II | -0.627330 | 0.000048 | 0.046098 |
| Longitudinal strain | CTSC | Cathepsin C / Dipeptidyl peptidase 1 | Inflammation | 0.691463 | 0.000016 | 0.046098 |

**Supplemental Table 2A:** Discrimination Indices for CTSC, NTproBNP, and hsTnT

|  | Concordance index (95% CI) | AUC at 6 months (95%CI) | AUC at 12 months  (95%CI) |
| --- | --- | --- | --- |
| Model 1* | 0.66 (0.60, 0.73) | 0.64 (0.55, 0.73) | 0.66 (0.59, 0.73) |
| Model 1 + CTSC | 0.68 (0.62, 0.74) | 0.68 (0.59, 0.77) | 0.68 (0.61, 0.75) |
| Model 1 + NTproBNP | 0.67 (0.61, 0.73) | 0.65 (0.56,0.73) | 0.66 (0.59, 0.73) |
| Model 1 + hsTnT | 0.66 (0.60, 0.73) | 0.63 (0.54,0.73) | 0.66 (0.59, 0.74) |
| Model 1 + NTproBNP + CTSC | 0.68 (0.62, 0.74) | 0.68 (0.59, 0.77) | 0.68 (0.61, 0.75) |

*Model 1: Cox regression model for the outcome of cardiac dysfunction, as defined by an absolute decline in left ventricular ejection fraction ≥10% to a value of <50%. Covariates in this model included: age, race (Black, White), cancer treatment (Dox, Tras, Dox+Tras), smoking status (never, former, current), body mass index, hypertension and diabetes.

75 events at a median time of 7 months (Q1, Q3 4, 12 months)

**Supplemental Table 2B:** Integrated Discrimination and Net Reclassification Indices for CTSC, NTproBNP, and hsTnT

|  | IDI at 6 months | cNRI at 6 months | IDI at 12 months | cNRI at 12 months |
| --- | --- | --- | --- | --- |
| Model 1* | Reference | Reference | Reference | Reference |
| Model 1 + CTSC | 0.006 (-0.004, 0.027) | 0.154 (-0.052, 0.303) | 0.014 (0.00, 0.057) | 0.104 (-0.049, 0.270) |
| Model 1 + NTproBNP | -0.001 (-0.003, 0.020) | 0.012 (-0.146, 0.208) | -0.001 (-0.006, 0.026) | 0.052 (-0.118, 0.173) |
| Model 1 + hsTnT | 0.000 (-0.003, 0.020) | 0.009 (-0.166, 0.203) | 0.003 (-0.002, 0.024) | 0.085 (-0.136, 0.210) |

*Model 1: Cox regression model for the outcome of cardiac dysfunction, as defined by an absolute decline in left ventricular ejection fraction ≥10% to a value of <50%. Covariates in this model included: age, race (Black, White), cancer treatment (Dox, Tras, Dox+Tras), smoking status (never, former, current), body mass index, hypertension, and diabetes.

75 events at a median time of 7 months (Q1, Q3 4, 12 months)

**Supplemental Table 2C:** Associations between Biomarkers and Cardiac Dysfunction

|  | Biomarker HR per each SD Increase (95%CI) | Nagelkerke R^2^ for the model |
| --- | --- | --- |
| Model 1 + CTSC | 0.73 (0.57, 0.93) | 0.061 |
| Model 1 + NTproBNP | 1.10 (0.86, 1.42) | 0.050 |
| Model 1 + hsTnT | 0.91 (0.71, 1.16) | 0.050 |

Hazard Ratio was reported per SD increase in biomarker levels. Model 1: Cox regression model for the cardiac dysfunction, adjusted for age, race (Black, White), cancer treatment (Dox, Tras, Dox+Tras), smoking status (never, former, current), body mass index, hypertension and diabetes.

75 events at a median time of 7 months (Q1, Q3 4, 12 months)

**Supplemental Table 3** Individual proteins associated with cardiac structure and function in the lagged analysis

| **Echocardiographic measure** | **Gene** | **Protein name** | **Panel** | **Beta** | **P value** | **FDR** |
| --- | --- | --- | --- | --- | --- | --- |
| E/e' | NPPC | C-type natriuretic peptide | Inflammation | 0.421616 | 0.000011 | 0.023273 |
| E/e' | ENDOU | Poly(U)-specific endoribonuclease | Oncology II | 0.516065 | 0.000016 | 0.023273 |
| LA volume index | EGF | Pro-epidermal growth factor | Inflammation | 0.723631 | 0.000027 | 0.039260 |
| LA volume index | GP5 | Platelet glycoprotein V | Inflammation II | 1.658499 | 0.000022 | 0.039260 |
| LV mass index | TMPRSS15 | Enteropeptidase | Oncology | -2.360104 | 0.000010 | 0.015082 |
| LV mass index | SERPINA9 | Serpin A9 | Oncology | -2.252492 | 0.000029 | 0.028326 |
| LV mass index | PROK1 | Prokineticin-1 | Inflammation | 2.609684 | 0.000001 | 0.001891 |
| Longitudinal strain | RASA1 | Ras GTPase-activating protein 1 | Neurology | -0.862655 | 0.000884 | 0.039266 |
| Longitudinal strain | CES3 | Carboxylesterase 3 | Oncology | -0.555158 | 0.000001 | 0.002348 |
| Longitudinal strain | PALM3 | Paralemmin-3 | Cardiometabolic II | -0.458709 | 0.001941 | 0.049612 |
| Longitudinal strain | PCARE | Photoreceptor cilium actin regulator | Neurology II | -0.402607 | 0.001137 | 0.042418 |
| Longitudinal strain | TSPAN7 | Tetraspanin-7 | Neurology II | -0.375280 | 0.001696 | 0.047026 |
| Longitudinal strain | LTA4H | Leukotriene A-4 hydrolase | Oncology | -0.358555 | 0.001297 | 0.043139 |
| Longitudinal strain | RNASE3 | Eosinophil cationic protein | Cardiometabolic | 0.148309 | 0.001573 | 0.045875 |
| Longitudinal strain | MNDA | Myeloid cell nuclear differentiation antigen | Cardiometabolic | 0.169475 | 0.001726 | 0.047038 |
| Longitudinal strain | EIF4E | Eukaryotic translation initiation factor 4E | Inflammation II | 0.192733 | 0.000752 | 0.038815 |
| Longitudinal strain | DNAJC9 | DnaJ homolog subfamily C member 9 | Oncology II | 0.198499 | 0.001405 | 0.043681 |
| Longitudinal strain | FOXO3 | Forkhead box protein O3 | Oncology | 0.200282 | 0.000495 | 0.038413 |
| Longitudinal strain | DNAJB1 | DnaJ homolog subfamily B member 1 | Oncology | 0.205514 | 0.000875 | 0.039266 |
| Longitudinal strain | CA2 | Carbonic anhydrase 2 | Neurology | 0.211913 | 0.001354 | 0.043681 |
| Longitudinal strain | DNAJC6 | Putative tyrosine-protein phosphatase auxilin | Cardiometabolic II | 0.214033 | 0.001762 | 0.047546 |
| Longitudinal strain | TBCA | Tubulin-specific chaperone A | Inflammation II | 0.216963 | 0.001136 | 0.042418 |
| Longitudinal strain | HAGH | Hydroxyacylglutathione hydrolase, mitochondrial | Oncology | 0.219973 | 0.000479 | 0.038413 |
| Longitudinal strain | PPME1 | Protein phosphatase methylesterase 1 | Oncology | 0.223183 | 0.000954 | 0.039467 |
| Longitudinal strain | SIRT2 | NAD-dependent protein deacetylase sirtuin-2 | Oncology | 0.228015 | 0.001188 | 0.042585 |
| Longitudinal strain | DTYMK | Thymidylate kinase | Cardiometabolic II | 0.231922 | 0.001034 | 0.040099 |
| Longitudinal strain | VTA1 | Vacuolar protein sorting-associated protein VTA1 homolog | Neurology | 0.239793 | 0.001922 | 0.049558 |
| Longitudinal strain | CD2AP | CD2-associated protein | Cardiometabolic | 0.240000 | 0.001270 | 0.043139 |
| Longitudinal strain | IGBP1 | Immunoglobulin-binding protein 1 | Neurology II | 0.240837 | 0.000963 | 0.039467 |
| Longitudinal strain | MIF | Macrophage migration inhibitory factor | Neurology | 0.243324 | 0.001174 | 0.042585 |
| Longitudinal strain | C9orf40 | Uncharacterized protein C9orf40 | Oncology II | 0.243573 | 0.000984 | 0.039467 |
| Longitudinal strain | ATG4A | Cysteine protease ATG4A | Oncology | 0.243915 | 0.000365 | 0.037666 |
| Longitudinal strain | AHSP | Alpha-hemoglobin-stabilizing protein | Neurology | 0.244176 | 0.000463 | 0.038413 |
| Longitudinal strain | MYL4 | Myosin light chain 4 | Cardiometabolic II | 0.247004 | 0.001839 | 0.048290 |
| Longitudinal strain | SH3GLB2 | Endophilin-B2 | Neurology II | 0.247874 | 0.001710 | 0.047026 |
| Longitudinal strain | RANBP1 | Ran-specific GTPase-activating protein | Cardiometabolic II | 0.249061 | 0.001623 | 0.045976 |
| Longitudinal strain | AKT1S1 | Proline-rich AKT1 substrate 1 | Neurology | 0.249850 | 0.000753 | 0.038815 |
| Longitudinal strain | AK1 | Adenylate kinase isoenzyme 1 | Cardiometabolic | 0.251599 | 0.000251 | 0.037666 |
| Longitudinal strain | UROD | Uroporphyrinogen decarboxylase | Inflammation II | 0.252247 | 0.001446 | 0.043952 |
| Longitudinal strain | USP25 | Ubiquitin carboxyl-terminal hydrolase 25 | Oncology II | 0.252629 | 0.001821 | 0.048248 |
| Longitudinal strain | PLPBP | Pyridoxal phosphate homeostasis protein | Cardiometabolic | 0.253145 | 0.000862 | 0.039266 |
| Longitudinal strain | EIF4EBP1 | Eukaryotic translation initiation factor 4E-binding protein 1 | Cardiometabolic | 0.253695 | 0.000198 | 0.037666 |
| Longitudinal strain | ATXN3 | Ataxin-3 | Neurology II | 0.256651 | 0.000878 | 0.039266 |
| Longitudinal strain | BLVRB | Flavin reductase (NADPH) | Neurology | 0.258644 | 0.000946 | 0.039467 |
| Longitudinal strain | PSMG4 | Proteasome assembly chaperone 4 | Inflammation II | 0.259197 | 0.000427 | 0.038413 |
| Longitudinal strain | AARSD1 | Alanyl-tRNA editing protein Aarsd1 | Oncology | 0.260734 | 0.000391 | 0.037666 |
| Longitudinal strain | GMPR2 | GMP reductase 2 | Inflammation II | 0.261519 | 0.001041 | 0.040099 |
| Longitudinal strain | CRYZL1 | Quinone oxidoreductase-like protein 1 | Cardiometabolic II | 0.264072 | 0.001407 | 0.043681 |
| Longitudinal strain | PSMD9 | 26S proteasome non-ATPase regulatory subunit 9 | Oncology | 0.264567 | 0.000535 | 0.038413 |
| Longitudinal strain | RILP | Rab-interacting lysosomal protein | Oncology | 0.264663 | 0.000361 | 0.037666 |
| Longitudinal strain | PIK3AP1 | Phosphoinositide 3-kinase adapter protein 1 | Inflammation | 0.265735 | 0.000193 | 0.037666 |
| Longitudinal strain | LARP1 | La-related protein 1 | Oncology II | 0.270693 | 0.001538 | 0.045329 |
| Longitudinal strain | EIF2S2 | Eukaryotic translation initiation factor 2 subunit 2 | Cardiometabolic II | 0.271326 | 0.001146 | 0.042418 |
| Longitudinal strain | ASRGL1 | Isoaspartyl peptidase/L-asparaginase | Cardiometabolic II | 0.272637 | 0.001600 | 0.045976 |
| Longitudinal strain | BECN1 | Beclin-1 | Cardiometabolic II | 0.274836 | 0.001314 | 0.043139 |
| Longitudinal strain | PARK7 | Parkinson disease protein 7 | Neurology | 0.275662 | 0.001483 | 0.044158 |
| Longitudinal strain | PEBP1 | Phosphatidylethanolamine-binding protein 1 | Neurology | 0.276690 | 0.000792 | 0.039054 |
| Longitudinal strain | RABGAP1L | Rab GTPase-activating protein 1-like | Inflammation | 0.276899 | 0.000297 | 0.037666 |
| Longitudinal strain | KYAT1 | Kynurenine--oxoglutarate transaminase 1 | Cardiometabolic | 0.279656 | 0.001797 | 0.048063 |
| Longitudinal strain | ACP1 | Low molecular weight phosphotyrosine protein phosphatase | Inflammation II | 0.279981 | 0.000284 | 0.037666 |
| Longitudinal strain | ACYP1 | Acylphosphatase-1 | Inflammation II | 0.281181 | 0.000717 | 0.038815 |
| Longitudinal strain | DDI2 | Protein DDI1 homolog 2 | Inflammation II | 0.285468 | 0.000335 | 0.037666 |
| Longitudinal strain | TRAF2 | TNF receptor-associated factor 2 | Inflammation | 0.287598 | 0.000599 | 0.038413 |
| Longitudinal strain | THTPA | Thiamine-triphosphatase | Oncology II | 0.290468 | 0.000573 | 0.038413 |
| Longitudinal strain | ST13 | Hsc70-interacting protein | Inflammation II | 0.291291 | 0.001194 | 0.042585 |
| Longitudinal strain | SNX15 | Sorting nexin-15 | Inflammation II | 0.292877 | 0.000829 | 0.039243 |
| Longitudinal strain | DFFA | DNA fragmentation factor subunit alpha | Inflammation | 0.293960 | 0.000546 | 0.038413 |
| Longitudinal strain | INPP5D | Phosphatidylinositol 3,4,5-trisphosphate 5-phosphatase 1 | Cardiometabolic II | 0.294559 | 0.000181 | 0.037666 |
| Longitudinal strain | TXN | Thioredoxin | Inflammation II | 0.296583 | 0.000123 | 0.037666 |
| Longitudinal strain | SOD1 | Superoxide dismutase [Cu-Zn] | Cardiometabolic | 0.298114 | 0.000766 | 0.038815 |
| Longitudinal strain | LHPP | Phospholysine phosphohistidine inorganic pyrophosphate phosphatase | Inflammation | 0.300056 | 0.000280 | 0.037666 |
| Longitudinal strain | DNAJB2 | DnaJ homolog subfamily B member 2 | Inflammation II | 0.300947 | 0.000484 | 0.038413 |
| Longitudinal strain | NUDT5 | ADP-sugar pyrophosphatase | Neurology | 0.302078 | 0.001628 | 0.045976 |
| Longitudinal strain | PSMG3 | Proteasome assembly chaperone 3 | Inflammation | 0.304118 | 0.000171 | 0.037666 |
| Longitudinal strain | BOLA2 / BOLA2B | BolA family member 2 / BolA Family Member 2B | Cardiometabolic II | 0.307338 | 0.000750 | 0.038815 |
| Longitudinal strain | CENPF | Centromere protein F | Oncology II | 0.312404 | 0.000060 | 0.034801 |
| Longitudinal strain | GGCT | Gamma-glutamylcyclotransferase | Cardiometabolic II | 0.313496 | 0.000629 | 0.038815 |
| Longitudinal strain | CCS | Copper chaperone for superoxide dismutase | Neurology | 0.314316 | 0.000587 | 0.038413 |
| Longitudinal strain | STAMBP | STAM-binding protein | Neurology | 0.314371 | 0.000984 | 0.039467 |
| Longitudinal strain | APRT | Adenine phosphoribosyltransferase | Neurology | 0.317647 | 0.001006 | 0.039789 |
| Longitudinal strain | IMPACT | Protein IMPACT | Neurology II | 0.318093 | 0.000294 | 0.037666 |
| Longitudinal strain | PRKAR2A | cAMP-dependent protein kinase type II-alpha regulatory subunit | Neurology II | 0.319433 | 0.001275 | 0.043139 |
| Longitudinal strain | ANKRD54 | Ankyrin repeat domain-containing protein 54 | Oncology | 0.320013 | 0.000143 | 0.037666 |
| Longitudinal strain | DNPH1 | 2'-deoxynucleoside 5'-phosphate N-hydrolase 1 | Inflammation | 0.323350 | 0.000308 | 0.037666 |
| Longitudinal strain | TGM2 | Protein-glutamine gamma-glutamyltransferase 2 | Cardiometabolic | 0.323580 | 0.000048 | 0.034617 |
| Longitudinal strain | AKR1B1 | Aldo-keto reductase family 1 member B1 | Oncology | 0.323767 | 0.000714 | 0.038815 |
| Longitudinal strain | HBQ1 | Hemoglobin subunit theta-1 | Oncology | 0.323873 | 0.000798 | 0.039054 |
| Longitudinal strain | CARHSP1 | Calcium-regulated heat-stable protein 1 | Neurology | 0.325153 | 0.000208 | 0.037666 |
| Longitudinal strain | PRDX2 | Peroxiredoxin-2 | Inflammation II | 0.326989 | 0.000478 | 0.038413 |
| Longitudinal strain | AMPD3 | AMP deaminase 3 | Neurology II | 0.331832 | 0.000352 | 0.037666 |
| Longitudinal strain | YJU2 | Splicing factor YJU2 | Oncology II | 0.332540 | 0.001272 | 0.043139 |
| Longitudinal strain | PRR5 | Proline-rich protein 5 | Inflammation II | 0.334096 | 0.001259 | 0.043139 |
| Longitudinal strain | ERI1 | 3'-5' exoribonuclease 1 | Oncology II | 0.335583 | 0.001471 | 0.044158 |
| Longitudinal strain | UBXN1 | UBX domain-containing protein 1 | Inflammation II | 0.339009 | 0.000137 | 0.037666 |
| Longitudinal strain | TBC1D17 | TBC1 domain family member 17 | Neurology | 0.341859 | 0.000373 | 0.037666 |
| Longitudinal strain | CHAC2 | Glutathione-specific gamma-glutamylcyclotransferase 2 | Oncology | 0.345476 | 0.000368 | 0.037666 |
| Longitudinal strain | CSNK2A1 | Casein kinase II subunit alpha | Neurology II | 0.350496 | 0.000732 | 0.038815 |
| Longitudinal strain | PKLR | Pyruvate kinase PKLR | Inflammation | 0.355814 | 0.000009 | 0.009139 |
| Longitudinal strain | TANK | TRAF family member-associated NF-kappa-B activator | Inflammation | 0.362116 | 0.000829 | 0.039243 |
| Longitudinal strain | TP53I3 | Quinone oxidoreductase PIG3 | Inflammation II | 0.364111 | 0.001302 | 0.043139 |
| Longitudinal strain | GCLM | Glutamate--cysteine ligase regulatory subunit | Cardiometabolic II | 0.364973 | 0.000264 | 0.037666 |
| Longitudinal strain | SNX9 | Sorting nexin-9 | Cardiometabolic | 0.370197 | 0.001371 | 0.043681 |
| Longitudinal strain | DXO | Decapping and exoribonuclease protein | Neurology II | 0.370378 | 0.000572 | 0.038413 |
| Longitudinal strain | NFX1 | Transcriptional repressor NF-X1 | Cardiometabolic II | 0.380365 | 0.000172 | 0.037666 |
| Longitudinal strain | RBKS | Ribokinase | Neurology | 0.383100 | 0.000312 | 0.037666 |
| Longitudinal strain | GET3 | ATPase GET3 | Cardiometabolic II | 0.388947 | 0.000695 | 0.038815 |
| Longitudinal strain | RIDA | 2-iminobutanoate/2-iminopropanoate deaminase | Inflammation II | 0.393247 | 0.001445 | 0.043952 |
| Longitudinal strain | PAGR1 | PAXIP1-associated glutamate-rich protein 1 | Cardiometabolic II | 0.398104 | 0.000557 | 0.038413 |
| Longitudinal strain | MRI1 | Methylthioribose-1-phosphate isomerase | Neurology II | 0.402622 | 0.000005 | 0.006671 |
| Longitudinal strain | ENOX2 | Ecto-NOX disulfide-thiol exchanger 2 | Cardiometabolic II | 0.405156 | 0.001640 | 0.045976 |
| Longitudinal strain | YWHAQ | 14-3-3 protein theta | Inflammation II | 0.414151 | 0.001867 | 0.048566 |
| Longitudinal strain | SYAP1 | Synapse-associated protein 1 | Inflammation II | 0.418973 | 0.000970 | 0.039467 |
| Longitudinal strain | GLOD4 | Glyoxalase domain-containing protein 4 | Inflammation | 0.432874 | 0.000646 | 0.038815 |
| Longitudinal strain | QDPR | Dihydropteridine reductase | Cardiometabolic | 0.443791 | 0.000437 | 0.038413 |
| Longitudinal strain | PSME1 | Proteasome activator complex subunit 1 | Neurology | 0.460033 | 0.000709 | 0.038815 |
| Longitudinal strain | PSME2 | Proteasome activator complex subunit 2 | Neurology | 0.465797 | 0.000567 | 0.038413 |
| Longitudinal strain | AAMDC | Mth938 domain-containing protein | Cardiometabolic II | 0.472653 | 0.000646 | 0.038815 |
| Longitudinal strain | THOP1 | Thimet oligopeptidase | Cardiometabolic | 0.484982 | 0.001404 | 0.043681 |
| Longitudinal strain | HDDC2 | 5'-deoxynucleotidase HDDC2 | Oncology II | 0.524877 | 0.000980 | 0.039467 |
| Longitudinal strain | UBAC1 | Ubiquitin-associated domain-containing protein 1 | Oncology | 0.572390 | 0.000388 | 0.037666 |

**Supplemental Table 4** Associations between identified proteomic markers and echocardiographic measures with interaction by cancer treatment

| **Time** | **Echocardiographic measure** | **Gene** | **Dox** | **Dox+Tras** | **Tras** | **P value** |
| --- | --- | --- | --- | --- | --- | --- |
| Contemporaneous | LVEF | CHEK2 | 0.606066 | 0.736468 | 0.290534 | 0.591049 |
| Contemporaneous | LVEF | HK2 | 1.035512 | 1.114604 | -0.007843 | 0.053973 |
| Contemporaneous | LVEF | SUSD1 | 0.731844 | 1.240600 | 0.336236 | 0.335733 |
| Contemporaneous | LVEF | IRAG2 | 0.415895 | 0.837528 | 0.025967 | 0.085490 |
| Contemporaneous | LVEF | SDC4 | 0.549502 | 0.297524 | 0.415627 | 0.750501 |
| Contemporaneous | LVEF | CEP164 | 0.770128 | 0.544723 | 0.847713 | 0.903549 |
| Contemporaneous | LVEF | EDAR | 0.863919 | 0.619324 | 0.425896 | 0.646802 |
| Contemporaneous | LVEF | ANXA11 | 1.425608 | 2.393203 | 0.261857 | 0.171875 |
| Contemporaneous | LVEF | CTSC | 1.218808 | 1.660228 | 0.772039 | 0.578374 |
| Contemporaneous | LVEF | ARID4B | 0.785429 | 0.734656 | 0.577773 | 0.882242 |
| Contemporaneous | LVEF | CASP1 | 0.329107 | 0.705828 | 0.057575 | 0.065509 |
| Contemporaneous | LVEF | ILKAP | 0.415398 | 1.031852 | 0.075314 | 0.057345 |
| Contemporaneous | LVEF | FMNL1 | 0.303739 | 0.656783 | 0.153959 | 0.178072 |
| Contemporaneous | LVEF | SKAP1 | 0.319481 | 0.506156 | 0.208962 | 0.563482 |
| Contemporaneous | LVEF | IMPA1 | 1.033998 | 1.366859 | 0.145591 | 0.119782 |
| Contemporaneous | LVEF | GPC5 | 1.118807 | 1.079608 | 0.794716 | 0.886175 |
| Contemporaneous | LVEF | GLB1 | 1.152066 | 1.042969 | 1.105991 | 0.990292 |
| Contemporaneous | LVEF | TNFSF14 | 0.907325 | 1.590874 | -0.131837 | 0.007717 |
| Contemporaneous | LVEF | CD63 | 0.681409 | 1.673079 | 0.399048 | 0.131135 |
| Contemporaneous | LVEF | BTC | 0.817725 | 0.878998 | 0.583035 | 0.851897 |
| Contemporaneous | LVEF | DDX58 | 0.454256 | 0.921769 | -0.088091 | 0.005320 |
| Contemporaneous | LVEF | ARHGAP25 | 0.537766 | 1.205584 | 0.391361 | 0.333409 |
| Contemporaneous | LVEF | FUS | 0.712248 | 1.468812 | 0.013084 | 0.035732 |
| Contemporaneous | LVEF | STX4 | 0.585942 | 1.049628 | 0.003730 | 0.086887 |
| Contemporaneous | LVEF | ARSB | 0.668484 | 1.199741 | 0.459338 | 0.468301 |
| Contemporaneous | LVEF | INPP1 | 0.871174 | 1.299917 | 0.227163 | 0.202187 |
| Contemporaneous | LVEF | HBEGF | 0.818094 | 1.091703 | 0.408617 | 0.420446 |
| Contemporaneous | LVEF | DDX39A | 0.942646 | 1.236924 | 0.438799 | 0.546689 |
| Contemporaneous | LVEF | GIMAP7 | 0.811194 | 0.966137 | 0.407319 | 0.621812 |
| Contemporaneous | LVEF | NMT1 | 0.639715 | 0.732803 | 0.142691 | 0.370764 |
| Contemporaneous | LVEF | RNASEH2A | 0.556352 | 1.239416 | 0.196327 | 0.160134 |
| Contemporaneous | LVEF | MORC3 | 0.514081 | 0.982554 | 0.277340 | 0.390740 |
| Contemporaneous | LVEF | C7orf50 | 0.653225 | 1.174812 | 0.413762 | 0.509042 |
| Contemporaneous | LVEF | ZHX2 | 0.636025 | 0.756542 | 0.230482 | 0.484592 |
| Contemporaneous | LVEF | PLSCR3 | 1.060321 | 2.488611 | 0.450201 | 0.100810 |
| Contemporaneous | LVEF | GIMAP8 | 0.556230 | 0.959587 | 0.154057 | 0.193552 |
| Contemporaneous | LVEF | SSH3 | 1.817342 | 2.301104 | 1.415963 | 0.853373 |
| Contemporaneous | LVEF | RCC1 | 0.655273 | 1.487364 | 0.409724 | 0.247429 |
| Contemporaneous | LVEF | VPS4B | 0.430311 | 0.959426 | 0.210993 | 0.186558 |
| Contemporaneous | LVEF | SH3BP1 | 0.645013 | 0.934143 | 0.300054 | 0.460209 |
| Contemporaneous | GLS | CTSC | 0.608331 | 1.030755 | 0.685588 | 0.626173 |
| Contemporaneous | GLS | KLK13 | -0.623216 | -0.773196 | -0.778493 | 0.893238 |
| Contemporaneous | GLS | S100A13 | -0.685245 | -0.336886 | -0.604593 | 0.794772 |
| Contemporaneous | LAVi | CTSC | -1.780762 | -2.093939 | -1.137664 | 0.758235 |
| Contemporaneous | LAVi | ILKAP | -0.613499 | -1.066872 | -0.809595 | 0.690997 |
| Contemporaneous | LAVi | GIMAP8 | -0.949467 | -0.971691 | -0.542980 | 0.700511 |
| Contemporaneous | LAVi | MNDA | -0.295936 | -0.683864 | -0.409716 | 0.440901 |
| Contemporaneous | LAVi | NADK | -0.433116 | -1.266982 | -0.924694 | 0.216563 |
| Contemporaneous | LAVi | AZU1 | -0.306859 | -1.018274 | -0.614373 | 0.205641 |
| Contemporaneous | LAVi | AMBN | -3.155229 | -2.267832 | -2.254869 | 0.774829 |
| Contemporaneous | LAVi | NCF2 | -0.339836 | -0.734111 | -0.349819 | 0.459519 |
| Contemporaneous | LAVi | HGF | -0.201574 | -1.652774 | -2.504794 | 0.002072 |
| Contemporaneous | LAVi | IL1RN | -0.993317 | -0.973603 | -0.963096 | 0.998733 |
| Contemporaneous | LAVi | FGR | -0.430347 | -0.979215 | -0.615510 | 0.330585 |
| Contemporaneous | LAVi | PADI4 | -0.367770 | -0.573837 | -0.439591 | 0.832489 |
| Contemporaneous | LAVi | CASP10 | -0.506277 | -0.976040 | -0.820220 | 0.522597 |
| Contemporaneous | LAVi | NPM1 | -0.518243 | -0.865007 | -0.465686 | 0.655628 |
| Contemporaneous | LAVi | CPPED1 | -0.324529 | -1.323854 | -0.824776 | 0.097632 |
| Contemporaneous | LAVi | MPO | -0.491535 | -1.343894 | -1.234537 | 0.208627 |
| Contemporaneous | LAVi | PQBP1 | -0.651887 | -1.090857 | -0.503780 | 0.643301 |
| Contemporaneous | LAVi | SRP14 | -0.540032 | -1.060430 | -0.475931 | 0.471992 |
| Contemporaneous | LAVi | ELOA | -0.581985 | -0.785532 | -0.540406 | 0.875261 |
| Contemporaneous | LAVi | APEX1 | -0.444805 | -1.049371 | -0.501168 | 0.287223 |
| Contemporaneous | LAVi | TOR1AIP1 | -0.387472 | -0.750209 | -0.623247 | 0.624868 |
| Contemporaneous | LAVi | AHNAK | -1.882176 | -0.223795 | -0.708707 | 0.143067 |
| Contemporaneous | LAVi | NAGK | -0.618930 | -1.457780 | -1.155321 | 0.388718 |
| Contemporaneous | LAVi | FOXJ3 | -0.730836 | -0.676308 | -0.624044 | 0.973802 |
| Contemporaneous | LAVi | MKI67 | -0.524497 | -0.882874 | -0.376580 | 0.581455 |
| Contemporaneous | LAVi | BAG4 | -0.593983 | -1.525784 | -0.653347 | 0.351058 |
| Contemporaneous | LAVi | BCL2L15 | -0.326047 | -1.441178 | -0.765971 | 0.077954 |
| Contemporaneous | LAVi | TOP2B | -0.487497 | -0.848187 | -0.482181 | 0.736556 |
| Contemporaneous | LAVi | PAXX | -0.724513 | -2.018652 | -0.955626 | 0.289161 |
| Contemporaneous | LAVi | KLF4 | -0.768877 | -1.017505 | -0.483716 | 0.651798 |
| Contemporaneous | LAVi | MRI1 | -0.464255 | -1.270322 | -0.680727 | 0.284032 |
| Contemporaneous | LAVi | SAFB2 | -0.937865 | -1.489228 | -0.652868 | 0.589397 |
| Contemporaneous | LAVi | SNRPB2 | -0.610059 | -0.625665 | -0.547025 | 0.984016 |
| Contemporaneous | LAVi | HDGFL2 | -0.524085 | -1.056163 | -0.512268 | 0.602419 |
| Contemporaneous | LAVi | KCTD5 | -0.816315 | -1.355200 | -0.895751 | 0.775343 |
| Contemporaneous | LAVi | BAP18 | -0.505434 | -0.839992 | -0.455323 | 0.722657 |
| Contemporaneous | LAVi | THAP12 | -0.556284 | -1.588698 | -0.440220 | 0.163010 |
| Contemporaneous | LAVi | ERI1 | -0.680577 | -1.286310 | -0.531958 | 0.559308 |
| Contemporaneous | LAVi | RPE | -0.618634 | -0.797836 | -1.113300 | 0.627282 |
| Contemporaneous | LAVi | RAB44 | -0.383661 | -0.793258 | -0.463215 | 0.596684 |
| Contemporaneous | LAVi | GPD1 | 1.026578 | 1.809317 | 0.020896 | 0.067097 |
| Contemporaneous | LAVi | RALY | -0.723012 | -1.260897 | -0.517915 | 0.492553 |
| Contemporaneous | LAVi | WDR46 | -0.700718 | -0.770085 | -0.548785 | 0.921111 |
| Contemporaneous | LAVi | TDP1 | -0.535931 | -0.892336 | -0.754777 | 0.758406 |
| Contemporaneous | LAVi | DUT | -0.365309 | -1.263752 | -0.758317 | 0.136624 |
| Contemporaneous | LAVi | SMNDC1 | -0.480991 | -0.777171 | -0.533593 | 0.788750 |
| Contemporaneous | LAVi | CWC15 | -0.717361 | -0.856958 | -0.631932 | 0.945460 |
| Lagged | GLS | AK1 | 0.033755 | 0.185734 | -0.231184 | 0.901391 |
| Lagged | GLS | SNX9 | 0.018927 | 0.399731 | -0.333351 | 0.863382 |
| Lagged | GLS | TGM2 | 0.011781 | 0.165884 | -0.143189 | 0.311259 |
| Lagged | GLS | PLPBP | 0.080641 | 0.298417 | -0.210145 | 0.974128 |
| Lagged | GLS | MNDA | 0.030624 | 0.131560 | -0.051813 | 0.376531 |
| Lagged | GLS | EIF4EBP1 | 0.012326 | 0.271661 | -0.217919 | 0.932237 |
| Lagged | GLS | QDPR | 0.000695 | 0.501061 | -0.253646 | 0.721576 |
| Lagged | GLS | CD2AP | -0.000503 | 0.260656 | -0.254416 | 0.916189 |
| Lagged | GLS | THOP1 | -0.064806 | 0.555155 | -0.121254 | 0.798282 |
| Lagged | GLS | RNASE3 | 0.064067 | -0.007116 | -0.045218 | 0.685576 |
| Lagged | GLS | KYAT1 | 0.064090 | 0.360850 | -0.166791 | 0.487273 |
| Lagged | GLS | SOD1 | -0.158209 | -0.023903 | -0.133474 | 0.875245 |
| Lagged | GLS | RABGAP1L | 0.021151 | 0.234795 | -0.252132 | 0.704347 |
| Lagged | GLS | TANK | -0.037068 | 0.256528 | -0.348874 | 0.777989 |
| Lagged | GLS | TRAF2 | 0.028951 | 0.369624 | -0.261145 | 0.971460 |
| Lagged | GLS | PSMG3 | 0.028031 | 0.202246 | -0.324345 | 0.848327 |
| Lagged | GLS | PIK3AP1 | 0.040919 | 0.248806 | -0.247464 | 0.867157 |
| Lagged | GLS | DFFA | 0.087089 | 0.476974 | -0.269711 | 0.966421 |
| Lagged | GLS | PKLR | 0.022973 | 0.296683 | -0.192967 | 0.993957 |
| Lagged | GLS | GLOD4 | 0.061447 | 0.424263 | -0.402565 | 0.962260 |
| Lagged | GLS | DNPH1 | 0.028978 | 0.227445 | -0.294929 | 0.832578 |
| Lagged | GLS | LHPP | -0.000869 | 0.226613 | -0.217548 | 0.983091 |
| Lagged | GLS | RASA1 | -0.108297 | -0.546479 | -0.079626 | 0.759805 |
| Lagged | GLS | TBC1D17 | 0.056782 | 0.224717 | -0.364435 | 0.963683 |
| Lagged | GLS | CARHSP1 | 0.047613 | 0.218522 | -0.305060 | 0.950588 |
| Lagged | GLS | AKT1S1 | 0.038527 | 0.245977 | -0.311804 | 0.921240 |
| Lagged | GLS | APRT | 0.028966 | 0.448700 | -0.378754 | 0.787436 |
| Lagged | GLS | STAMBP | 0.055714 | 0.381488 | -0.342756 | 0.900086 |
| Lagged | GLS | PSME1 | 0.050882 | 0.548237 | -0.494145 | 0.995395 |
| Lagged | GLS | CCS | 0.061335 | 0.217280 | -0.334055 | 0.989632 |
| Lagged | GLS | RBKS | -0.034105 | 0.504585 | -0.315506 | 0.433288 |
| Lagged | GLS | PSME2 | 0.016255 | 0.372487 | -0.532168 | 0.867357 |
| Lagged | GLS | VTA1 | 0.043767 | 0.246030 | -0.264386 | 0.726673 |
| Lagged | GLS | MIF | 0.052632 | 0.147409 | -0.255796 | 0.740138 |
| Lagged | GLS | AHSP | 0.022082 | 0.166895 | -0.234249 | 0.721503 |
| Lagged | GLS | PEBP1 | 0.005428 | 0.120621 | -0.289523 | 0.397267 |
| Lagged | GLS | NUDT5 | -0.020674 | 0.262941 | -0.365725 | 0.530914 |
| Lagged | GLS | BLVRB | 0.020478 | 0.118844 | -0.309259 | 0.877461 |
| Lagged | GLS | CA2 | 0.015086 | 0.056231 | -0.265767 | 0.671173 |
| Lagged | GLS | PARK7 | 0.037642 | 0.150882 | -0.302537 | 0.675597 |
| Lagged | GLS | ANKRD54 | -0.015754 | 0.310949 | -0.340639 | 0.873400 |
| Lagged | GLS | CHAC2 | 0.076265 | 0.402937 | -0.222857 | 0.982734 |
| Lagged | GLS | AKR1B1 | 0.159729 | 0.147022 | -0.074210 | 0.935223 |
| Lagged | GLS | ATG4A | 0.033545 | 0.147656 | -0.267442 | 0.940897 |
| Lagged | GLS | FOXO3 | 0.035101 | 0.207137 | -0.138487 | 0.929939 |
| Lagged | GLS | PSMD9 | 0.005929 | 0.218115 | -0.279706 | 0.972214 |
| Lagged | GLS | AARSD1 | 0.024910 | 0.294297 | -0.301275 | 0.833919 |
| Lagged | GLS | UBAC1 | -0.054994 | 0.579482 | -0.566947 | 0.853790 |
| Lagged | GLS | CES3 | -0.300259 | -0.228169 | -0.409422 | 0.319469 |
| Lagged | GLS | PPME1 | 0.008208 | 0.209424 | -0.301200 | 0.738219 |
| Lagged | GLS | DNAJB1 | 0.001588 | 0.160526 | -0.236458 | 0.955147 |
| Lagged | GLS | SIRT2 | 0.039678 | 0.293574 | -0.264742 | 0.992743 |
| Lagged | GLS | HBQ1 | 0.072806 | 0.398551 | -0.307330 | 0.920847 |
| Lagged | GLS | RILP | 0.033539 | 0.252231 | -0.290591 | 0.847054 |
| Lagged | GLS | LTA4H | 0.024544 | 0.048556 | -0.398506 | 0.516053 |
| Lagged | GLS | HAGH | 0.024916 | 0.214851 | -0.233488 | 0.868490 |
| Lagged | GLS | GCLM | 0.017804 | 0.007629 | -0.186522 | 0.320658 |
| Lagged | GLS | ENOX2 | 0.037188 | 0.162932 | -0.425345 | 0.940662 |
| Lagged | GLS | NFX1 | -0.011203 | 0.116134 | -0.149393 | 0.977755 |
| Lagged | GLS | PALM3 | -0.274484 | -0.426169 | -0.149542 | 0.939873 |
| Lagged | GLS | PAGR1 | 0.085305 | 0.162102 | -0.376257 | 0.482765 |
| Lagged | GLS | BECN1 | -0.000697 | 0.176602 | -0.285435 | 0.841197 |
| Lagged | GLS | AAMDC | 0.074593 | 0.186320 | -0.436439 | 0.766200 |
| Lagged | GLS | MYL4 | -0.013450 | 0.156592 | -0.279038 | 0.933634 |
| Lagged | GLS | DNAJC6 | 0.003043 | 0.188015 | -0.220913 | 0.956033 |
| Lagged | GLS | RANBP1 | -0.010480 | 0.017321 | -0.285710 | 0.746076 |
| Lagged | GLS | INPP5D | 0.022849 | 0.074301 | -0.291297 | 0.652344 |
| Lagged | GLS | BOLA2_BOLA2B | 0.043514 | 0.125042 | -0.321775 | 0.862445 |
| Lagged | GLS | EIF2S2 | 0.030135 | 0.142715 | -0.262836 | 0.534197 |
| Lagged | GLS | CRYZL1 | 0.028650 | 0.132940 | -0.271993 | 0.843651 |
| Lagged | GLS | ASRGL1 | 0.048656 | 0.328844 | -0.225916 | 0.597573 |
| Lagged | GLS | GET3 | 0.057710 | 0.121358 | -0.008021 | 0.490695 |
| Lagged | GLS | GGCT | 0.083535 | 0.167059 | -0.319435 | 0.967462 |
| Lagged | GLS | DTYMK | 0.042046 | 0.106806 | -0.249892 | 0.955545 |
| Lagged | GLS | PRR5 | 0.013938 | 0.452344 | -0.262145 | 0.810989 |
| Lagged | GLS | PSMG4 | 0.028319 | 0.071929 | -0.291447 | 0.795711 |
| Lagged | GLS | ACP1 | 0.079024 | 0.151763 | -0.246607 | 0.631654 |
| Lagged | GLS | EIF4E | 0.013814 | 0.045528 | -0.192594 | 0.853818 |
| Lagged | GLS | DDI2 | -0.001316 | -0.045486 | -0.302471 | 0.772520 |
| Lagged | GLS | UROD | 0.033672 | 0.132074 | -0.271834 | 0.967191 |
| Lagged | GLS | YWHAQ | 0.040355 | 0.377083 | -0.506467 | 0.734036 |
| Lagged | GLS | TBCA | 0.042962 | 0.149025 | -0.231586 | 0.824312 |
| Lagged | GLS | GMPR2 | 0.028518 | 0.109220 | -0.306213 | 0.907584 |
| Lagged | GLS | ST13 | 0.036282 | -0.026067 | -0.340038 | 0.862397 |
| Lagged | GLS | TP53I3 | 0.046817 | 0.261075 | -0.368524 | 0.927583 |
| Lagged | GLS | RIDA | -0.096440 | 0.352127 | -0.234761 | 0.415173 |
| Lagged | GLS | SYAP1 | 0.166962 | 0.257693 | -0.492313 | 0.660247 |
| Lagged | GLS | ACYP1 | 0.064811 | 0.176374 | -0.278796 | 0.872823 |
| Lagged | GLS | SNX15 | 0.031166 | 0.122294 | -0.293033 | 0.891463 |
| Lagged | GLS | UBXN1 | 0.081421 | 0.138026 | -0.219369 | 0.944740 |
| Lagged | GLS | DNAJB2 | 0.060240 | 0.088807 | -0.290314 | 0.934588 |
| Lagged | GLS | PRDX2 | 0.043162 | 0.022996 | -0.356209 | 0.907930 |
| Lagged | GLS | TXN | 0.114306 | 0.061568 | -0.273373 | 0.997242 |
| Lagged | GLS | TSPAN7 | -0.093481 | -0.438976 | -0.186270 | 0.854057 |
| Lagged | GLS | PCARE | 0.011860 | -0.267054 | -0.114676 | 0.918183 |
| Lagged | GLS | MRI1 | 0.058670 | 0.206008 | -0.187943 | 0.447597 |
| Lagged | GLS | CSNK2A1 | 0.004601 | 0.057335 | -0.300918 | 0.834539 |
| Lagged | GLS | IGBP1 | 0.019062 | 0.018097 | -0.250916 | 0.759240 |
| Lagged | GLS | DXO | 0.133284 | 0.322001 | -0.432878 | 0.746722 |
| Lagged | GLS | IMPACT | 0.053981 | 0.017783 | -0.305035 | 0.840282 |
| Lagged | GLS | PRKAR2A | 0.026034 | 0.175372 | -0.256123 | 0.572194 |
| Lagged | GLS | AMPD3 | 0.048517 | 0.045133 | -0.359409 | 0.854196 |
| Lagged | GLS | SH3GLB2 | 0.045827 | 0.080285 | -0.319096 | 0.849032 |
| Lagged | GLS | ATXN3 | 0.016671 | -0.036216 | -0.302689 | 0.725944 |
| Lagged | GLS | ERI1 | 0.083780 | 0.282603 | -0.214381 | 0.703602 |
| Lagged | GLS | CENPF | 0.045413 | 0.105205 | -0.369546 | 0.655944 |
| Lagged | GLS | LARP1 | 0.028753 | 0.197299 | -0.336512 | 0.926616 |
| Lagged | GLS | THTPA | 0.060192 | 0.184696 | -0.291893 | 0.473923 |
| Lagged | GLS | YJU2 | 0.024613 | 0.248029 | -0.284597 | 0.985786 |
| Lagged | GLS | USP25 | 0.034716 | 0.095428 | -0.271822 | 0.973656 |
| Lagged | GLS | HDDC2 | 0.097612 | 0.344373 | -0.550591 | 0.842150 |
| Lagged | GLS | DNAJC9 | 0.041973 | 0.032505 | -0.223094 | 0.865136 |
| Lagged | GLS | C9orf40 | 0.034481 | -0.066694 | -0.301298 | 0.637017 |
| Lagged | E/e' | NPPC | 0.115688 | 0.473269 | 0.134114 | 0.048420 |
| Lagged | E/e' | ENDOU | 0.282768 | 0.371637 | 0.501849 | 0.024894 |
| Lagged | LVMi | PROK1 | -0.256583 | -0.403609 | 1.252793 | 0.012932 |
| Lagged | LVMi | SERPINA9 | 0.090044 | 1.583212 | -1.762254 | 0.558982 |
| Lagged | LVMi | TMPRSS15 | -0.612312 | -1.000195 | -1.190648 | 0.779836 |
| Lagged | LAVi | EGF | 0.160343 | -0.351519 | -0.192990 | 0.466708 |
| Lagged | LAVi | GP5 | 0.134171 | -1.306633 | -0.977964 | 0.761879 |

P values are for the interaction term between identified proteomic markers and cancer treatment. Raw p-values < 0.05/210 were considered statistically significant. LVEF: left ventricular ejection fraction GLS: global longitudinal strain LVMi: left ventricular mass index LAVi: left atrial volume index

**Supplemental Table 5** Associations between identified metabolomic markers and echocardiographic measures with interaction by cancer treatment

| **Time** | **Echocardiographic measure** | **Metabolite** | **Dox** | **Dox+Tras** | **Tras** | **P value** |
| --- | --- | --- | --- | --- | --- | --- |
| Contemporaneous | LVEF | N-Acetylglutamine | -0.519739 | -0.085967 | -1.112703 | 0.103263 |
| Contemporaneous | LVEF | Alanine; Sarcosine | -0.975290 | -1.347973 | -1.122327 | 0.899170 |
| Contemporaneous | LVEF | Amino(iso)butyric acid; Dimethylglycine | 1.150825 | 1.159236 | 0.187050 | 0.186210 |
| Contemporaneous | LVEF | Aspartic acid | -0.820695 | -0.755683 | -1.249695 | 0.608105 |
| Contemporaneous | LVEF | Ala-Ala | -0.760786 | -0.404907 | -0.692199 | 0.581856 |
| Contemporaneous | LVEF | Pro-Gly | -0.819276 | -0.203714 | -1.116599 | 0.432707 |
| Contemporaneous | LVEF | Acetylasparagine | -1.153700 | -1.081746 | -0.848376 | 0.830403 |
| Contemporaneous | GLS | N-Acetylglutamine | -0.463946 | -0.331311 | -0.469520 | 0.887563 |
| Contemporaneous | GLS | Aspartic acid | -0.586367 | -0.661471 | -0.587311 | 0.978568 |
| Contemporaneous | GLS | Ala-Ala | -0.447306 | -0.392284 | -0.271878 | 0.576277 |
| Contemporaneous | GLS | Pro-Gly | -0.762410 | -0.515337 | -0.314094 | 0.364814 |
| Contemporaneous | GLS | Acetylasparagine | -0.678755 | -1.035098 | -0.236334 | 0.176227 |
| Contemporaneous | GLS | Bilirubin | 0.532980 | 0.282887 | 0.560434 | 0.778921 |
| Contemporaneous | GLS | TG(57:7) | -0.481172 | -0.993778 | -0.279703 | 0.226771 |
| Contemporaneous | LAVi | Perfluorooctanesulfonic acid (PFOS) | 1.716263 | 0.443107 | 1.338614 | 0.325578 |
| Lagged | GLS | SM(36:2;O2) | -0.670340 | -0.171956 | -0.338407 | 0.782160 |
| Lagged | GLS | 22:1-Glc-Cholesterol | -1.052151 | 0.170620 | 0.154498 | 0.687658 |
| Lagged | LVMi | Oleamide | 4.137113 | 3.539793 | -1.101363 | 0.895918 |
| Lagged | LVMi | Aspartic acid | 1.764815 | -0.439489 | 1.434389 | 0.407016 |
| Lagged | LVMi | Acetylasparagine | 2.182353 | 0.375874 | 1.550641 | 0.098125 |
| Lagged | LVMi | Putative Known Oxylipin C18H36O4 | -0.944364 | -2.616912 | -0.451200 | 0.525989 |
| Lagged | LAVi | DG(36:4) | -1.402014 | 1.515759 | 1.231776 | 0.569038 |
| Lagged | LAVi | PC(38:3;O) | 0.828734 | 2.194210 | -0.326060 | 0.066743 |
| Lagged | LAVi | Perfluorooctanesulfonic acid (PFOS) | 1.716263 | 0.443107 | 1.338614 | 0.639053 |

P values are for the interaction term between identified metabolomic markers and cancer treatment. Raw p-values < 0.05/24 were considered statistically significant. LVEF: left ventricular ejection fraction GLS: global longitudinal strain LVMi: left ventricular mass index LAVi: left atrial volume index

**Supplemental Table 6** Associations between identified proteomic markers and echocardiographic measures with interaction by race

| **Time** | **Echocardiographic measure** | **Gene** | **P value** |
| --- | --- | --- | --- |
| Contemporaneous | LVEF | CHEK2 | 0.708440 |
| Contemporaneous | LVEF | HK2 | 0.142228 |
| Contemporaneous | LVEF | SUSD1 | 0.898495 |
| Contemporaneous | LVEF | IRAG2 | 0.785501 |
| Contemporaneous | LVEF | SDC4 | 0.615613 |
| Contemporaneous | LVEF | CEP164 | 0.939664 |
| Contemporaneous | LVEF | EDAR | 0.734234 |
| Contemporaneous | LVEF | ANXA11 | 0.987302 |
| Contemporaneous | LVEF | CTSC | 0.294864 |
| Contemporaneous | LVEF | ARID4B | 0.550510 |
| Contemporaneous | LVEF | CASP1 | 0.600723 |
| Contemporaneous | LVEF | ILKAP | 0.123402 |
| Contemporaneous | LVEF | FMNL1 | 0.559825 |
| Contemporaneous | LVEF | SKAP1 | 0.961866 |
| Contemporaneous | LVEF | IMPA1 | 0.519653 |
| Contemporaneous | LVEF | GPC5 | 0.815871 |
| Contemporaneous | LVEF | GLB1 | 0.961240 |
| Contemporaneous | LVEF | TNFSF14 | 0.993880 |
| Contemporaneous | LVEF | CD63 | 0.535502 |
| Contemporaneous | LVEF | BTC | 0.498846 |
| Contemporaneous | LVEF | DDX58 | 0.318849 |
| Contemporaneous | LVEF | ARHGAP25 | 0.390703 |
| Contemporaneous | LVEF | FUS | 0.875953 |
| Contemporaneous | LVEF | STX4 | 0.400176 |
| Contemporaneous | LVEF | ARSB | 0.511772 |
| Contemporaneous | LVEF | INPP1 | 0.832182 |
| Contemporaneous | LVEF | HBEGF | 0.350008 |
| Contemporaneous | LVEF | DDX39A | 0.712844 |
| Contemporaneous | LVEF | GIMAP7 | 0.252621 |
| Contemporaneous | LVEF | NMT1 | 0.129786 |
| Contemporaneous | LVEF | RNASEH2A | 0.321161 |
| Contemporaneous | LVEF | MORC3 | 0.436704 |
| Contemporaneous | LVEF | C7orf50 | 0.836804 |
| Contemporaneous | LVEF | ZHX2 | 0.910794 |
| Contemporaneous | LVEF | PLSCR3 | 0.534055 |
| Contemporaneous | LVEF | GIMAP8 | 0.423449 |
| Contemporaneous | LVEF | SSH3 | 0.681322 |
| Contemporaneous | LVEF | RCC1 | 0.581643 |
| Contemporaneous | LVEF | VPS4B | 0.596904 |
| Contemporaneous | LVEF | SH3BP1 | 0.564111 |
| Contemporaneous | GLS | CTSC | 0.468494 |
| Contemporaneous | GLS | KLK13 | 0.485465 |
| Contemporaneous | GLS | S100A13 | 0.717271 |
| Contemporaneous | LAVi | CTSC | 0.156940 |
| Contemporaneous | LAVi | ILKAP | 0.533032 |
| Contemporaneous | LAVi | GIMAP8 | 0.880456 |
| Contemporaneous | LAVi | MNDA | 0.630026 |
| Contemporaneous | LAVi | NADK | 0.329441 |
| Contemporaneous | LAVi | AZU1 | 0.447469 |
| Contemporaneous | LAVi | AMBN | 0.531972 |
| Contemporaneous | LAVi | NCF2 | 0.739599 |
| Contemporaneous | LAVi | HGF | 0.466188 |
| Contemporaneous | LAVi | IL1RN | 0.721562 |
| Contemporaneous | LAVi | FGR | 0.649300 |
| Contemporaneous | LAVi | PADI4 | 0.532561 |
| Contemporaneous | LAVi | CASP10 | 0.347887 |
| Contemporaneous | LAVi | NPM1 | 0.334856 |
| Contemporaneous | LAVi | CPPED1 | 0.283933 |
| Contemporaneous | LAVi | MPO | 0.139004 |
| Contemporaneous | LAVi | PQBP1 | 0.721738 |
| Contemporaneous | LAVi | SRP14 | 0.202224 |
| Contemporaneous | LAVi | ELOA | 0.308975 |
| Contemporaneous | LAVi | APEX1 | 0.454458 |
| Contemporaneous | LAVi | TOR1AIP1 | 0.499447 |
| Contemporaneous | LAVi | AHNAK | 0.631689 |
| Contemporaneous | LAVi | NAGK | 0.487045 |
| Contemporaneous | LAVi | FOXJ3 | 0.665908 |
| Contemporaneous | LAVi | MKI67 | 0.131812 |
| Contemporaneous | LAVi | BAG4 | 0.227001 |
| Contemporaneous | LAVi | BCL2L15 | 0.744598 |
| Contemporaneous | LAVi | TOP2B | 0.866308 |
| Contemporaneous | LAVi | PAXX | 0.104288 |
| Contemporaneous | LAVi | KLF4 | 0.494962 |
| Contemporaneous | LAVi | MRI1 | 0.474701 |
| Contemporaneous | LAVi | SAFB2 | 0.258294 |
| Contemporaneous | LAVi | SNRPB2 | 0.394983 |
| Contemporaneous | LAVi | HDGFL2 | 0.480870 |
| Contemporaneous | LAVi | KCTD5 | 0.317021 |
| Contemporaneous | LAVi | BAP18 | 0.684733 |
| Contemporaneous | LAVi | THAP12 | 0.547371 |
| Contemporaneous | LAVi | ERI1 | 0.099019 |
| Contemporaneous | LAVi | RPE | 0.333621 |
| Contemporaneous | LAVi | RAB44 | 0.800908 |
| Contemporaneous | LAVi | GPD1 | 0.344133 |
| Contemporaneous | LAVi | RALY | 0.251279 |
| Contemporaneous | LAVi | WDR46 | 0.294401 |
| Contemporaneous | LAVi | TDP1 | 0.359431 |
| Contemporaneous | LAVi | DUT | 0.525286 |
| Contemporaneous | LAVi | SMNDC1 | 0.886064 |
| Contemporaneous | LAVi | CWC15 | 0.654886 |
| Lagged | GLS | AK1 | 0.747567 |
| Lagged | GLS | SNX9 | 0.589045 |
| Lagged | GLS | TGM2 | 0.282233 |
| Lagged | GLS | PLPBP | 0.616905 |
| Lagged | GLS | MNDA | 0.069102 |
| Lagged | GLS | EIF4EBP1 | 0.956138 |
| Lagged | GLS | QDPR | 0.535928 |
| Lagged | GLS | CD2AP | 0.556914 |
| Lagged | GLS | THOP1 | 0.703142 |
| Lagged | GLS | RNASE3 | 0.160072 |
| Lagged | GLS | KYAT1 | 0.717043 |
| Lagged | GLS | SOD1 | 0.851818 |
| Lagged | GLS | RABGAP1L | 0.659189 |
| Lagged | GLS | TANK | 0.417744 |
| Lagged | GLS | TRAF2 | 0.394259 |
| Lagged | GLS | PSMG3 | 0.878815 |
| Lagged | GLS | PIK3AP1 | 0.978870 |
| Lagged | GLS | DFFA | 0.241566 |
| Lagged | GLS | PKLR | 0.831559 |
| Lagged | GLS | GLOD4 | 0.628203 |
| Lagged | GLS | DNPH1 | 0.834183 |
| Lagged | GLS | LHPP | 0.684592 |
| Lagged | GLS | RASA1 | 0.187450 |
| Lagged | GLS | TBC1D17 | 0.814824 |
| Lagged | GLS | CARHSP1 | 0.793613 |
| Lagged | GLS | AKT1S1 | 0.990729 |
| Lagged | GLS | APRT | 0.931416 |
| Lagged | GLS | STAMBP | 0.932394 |
| Lagged | GLS | PSME1 | 0.803558 |
| Lagged | GLS | CCS | 0.857243 |
| Lagged | GLS | RBKS | 0.983410 |
| Lagged | GLS | PSME2 | 0.904687 |
| Lagged | GLS | VTA1 | 0.901233 |
| Lagged | GLS | MIF | 0.812972 |
| Lagged | GLS | AHSP | 0.713863 |
| Lagged | GLS | PEBP1 | 0.998770 |
| Lagged | GLS | NUDT5 | 0.807988 |
| Lagged | GLS | BLVRB | 0.679110 |
| Lagged | GLS | CA2 | 0.473198 |
| Lagged | GLS | PARK7 | 0.918739 |
| Lagged | GLS | ANKRD54 | 0.701623 |
| Lagged | GLS | CHAC2 | 0.478955 |
| Lagged | GLS | AKR1B1 | 0.374385 |
| Lagged | GLS | ATG4A | 0.782925 |
| Lagged | GLS | FOXO3 | 0.312304 |
| Lagged | GLS | PSMD9 | 0.815225 |
| Lagged | GLS | AARSD1 | 0.842844 |
| Lagged | GLS | UBAC1 | 0.717253 |
| Lagged | GLS | CES3 | 0.545726 |
| Lagged | GLS | PPME1 | 0.908736 |
| Lagged | GLS | DNAJB1 | 0.994496 |
| Lagged | GLS | SIRT2 | 0.854982 |
| Lagged | GLS | HBQ1 | 0.368973 |
| Lagged | GLS | RILP | 0.870687 |
| Lagged | GLS | LTA4H | 0.769955 |
| Lagged | GLS | HAGH | 0.588356 |
| Lagged | GLS | GCLM | 0.886005 |
| Lagged | GLS | ENOX2 | 0.351807 |
| Lagged | GLS | NFX1 | 0.632935 |
| Lagged | GLS | PALM3 | 0.408108 |
| Lagged | GLS | PAGR1 | 0.313356 |
| Lagged | GLS | BECN1 | 0.767471 |
| Lagged | GLS | AAMDC | 0.623420 |
| Lagged | GLS | MYL4 | 0.698500 |
| Lagged | GLS | DNAJC6 | 0.664345 |
| Lagged | GLS | RANBP1 | 0.832216 |
| Lagged | GLS | INPP5D | 0.624007 |
| Lagged | GLS | BOLA2_BOLA2B | 0.986168 |
| Lagged | GLS | EIF2S2 | 0.537377 |
| Lagged | GLS | CRYZL1 | 0.535348 |
| Lagged | GLS | ASRGL1 | 0.418270 |
| Lagged | GLS | GET3 | 0.979857 |
| Lagged | GLS | GGCT | 0.772297 |
| Lagged | GLS | DTYMK | 0.990170 |
| Lagged | GLS | PRR5 | 0.377506 |
| Lagged | GLS | PSMG4 | 0.973294 |
| Lagged | GLS | ACP1 | 0.480369 |
| Lagged | GLS | EIF4E | 0.827370 |
| Lagged | GLS | DDI2 | 0.571771 |
| Lagged | GLS | UROD | 0.980749 |
| Lagged | GLS | YWHAQ | 0.963330 |
| Lagged | GLS | TBCA | 0.879468 |
| Lagged | GLS | GMPR2 | 0.984874 |
| Lagged | GLS | ST13 | 0.721375 |
| Lagged | GLS | TP53I3 | 0.705060 |
| Lagged | GLS | RIDA | 0.788698 |
| Lagged | GLS | SYAP1 | 0.945981 |
| Lagged | GLS | ACYP1 | 0.954632 |
| Lagged | GLS | SNX15 | 0.983070 |
| Lagged | GLS | UBXN1 | 0.831768 |
| Lagged | GLS | DNAJB2 | 0.972681 |
| Lagged | GLS | PRDX2 | 0.324494 |
| Lagged | GLS | TXN | 0.868905 |
| Lagged | GLS | TSPAN7 | 0.143320 |
| Lagged | GLS | PCARE | 0.092209 |
| Lagged | GLS | MRI1 | 0.960539 |
| Lagged | GLS | CSNK2A1 | 0.974392 |
| Lagged | GLS | IGBP1 | 0.905236 |
| Lagged | GLS | DXO | 0.583301 |
| Lagged | GLS | IMPACT | 0.948653 |
| Lagged | GLS | PRKAR2A | 0.166212 |
| Lagged | GLS | AMPD3 | 0.748614 |
| Lagged | GLS | SH3GLB2 | 0.827396 |
| Lagged | GLS | ATXN3 | 0.942571 |
| Lagged | GLS | ERI1 | 0.168743 |
| Lagged | GLS | CENPF | 0.987594 |
| Lagged | GLS | LARP1 | 0.828051 |
| Lagged | GLS | THTPA | 0.738299 |
| Lagged | GLS | YJU2 | 0.827203 |
| Lagged | GLS | USP25 | 0.727434 |
| Lagged | GLS | HDDC2 | 0.495995 |
| Lagged | GLS | DNAJC9 | 0.681209 |
| Lagged | GLS | C9orf40 | 0.575562 |
| Lagged | E/e' | NPPC | 0.364221 |
| Lagged | E/e' | ENDOU | 0.938121 |
| Lagged | LVMi | PROK1 | 0.138744 |
| Lagged | LVMi | SERPINA9 | 0.111849 |
| Lagged | LVMi | TMPRSS15 | 0.502473 |
| Lagged | LAVi | EGF | 0.217818 |
| Lagged | LAVi | GP5 | 0.293601 |

P values are for the interaction term between identified proteomic markers and race (Black versus White). Raw p-values < 0.05/210 were considered statistically significant. LVEF: left ventricular ejection fraction GLS: global longitudinal strain LVMi: left ventricular mass index LAVi: left atrial volume index

**Supplemental Table 7** Associations between identified metabolomic markers and echocardiographic measures with interaction by race

| **Time** | **Echocardiographic measure** | **Metabolite** | **P value** |
| --- | --- | --- | --- |
| Contemporaneous | LVEF | N-Acetylglutamine | 0.079593 |
| Contemporaneous | LVEF | Alanine; Sarcosine | 0.920560 |
| Contemporaneous | LVEF | Amino(iso)butyric acid; Dimethylglycine | 0.733612 |
| Contemporaneous | LVEF | Aspartic acid | 0.206371 |
| Contemporaneous | LVEF | Ala-Ala | 0.282724 |
| Contemporaneous | LVEF | Pro-Gly | 0.060545 |
| Contemporaneous | LVEF | Acetylasparagine | 0.367441 |
| Contemporaneous | GLS | N-Acetylglutamine | 0.495882 |
| Contemporaneous | GLS | Aspartic acid | 0.765332 |
| Contemporaneous | GLS | Ala-Ala | 0.747000 |
| Contemporaneous | GLS | Pro-Gly | 0.466436 |
| Contemporaneous | GLS | Acetylasparagine | 0.514254 |
| Contemporaneous | GLS | Bilirubin | 0.315782 |
| Contemporaneous | GLS | TG(57:7 | 0.358091 |
| Contemporaneous | LAVi | Perfluorooctanesulfonic acid (PFOS) | 0.812419 |
| Lagged | GLS | SM(36:2;O2) | 0.671114 |
| Lagged | GLS | 22:1-Glc-Cholesterol | 0.312451 |
| Lagged | LVMi | Oleamide | 0.947187 |
| Lagged | LVMi | Aspartic acid | 0.110356 |
| Lagged | LVMi | Acetylasparagine | 0.175875 |
| Lagged | LVMi | Putative Known Oxylipin C18H36O4 | 0.658973 |
| Lagged | LAVi | DG(36:4) | 0.626712 |
| Lagged | LAVi | PC(38:3;O) | 0.581746 |
| Lagged | LAVi | Perfluorooctanesulfonic acid (PFOS) | 0.698334 |

P values are for the interaction term between identified metabolomic markers and race (Black versus White). Raw p-values < 0.05/24 were considered statistically significant. LVEF: left ventricular ejection fraction GLS: global longitudinal strain LVMi: left ventricular mass index LAVi: left atrial volume index

**Supplemental Table 8** Proteins associated with cardiac function corresponding to statistically enriched pathways

| **Category** | **Pathway** | **Enrichment FDR** | **Count** | **Proteins** |
| --- | --- | --- | --- | --- |
| BP | Protein deubiquitination | 0.01158965 | 7 | TANK/STAMBP/PARK7/UBXN1/DNAJB2/ATXN3/USP25 |
| BP | Protein modification by small protein removal | 0.01158965 | 7 | TANK/STAMBP/PARK7/UBXN1/DNAJB2/ATXN3/USP25 |
| BP | Macromolecule catabolic process | 0.02418080 | 29 | CHEK2/CTSC/GLB1/FUS/RNASEH2A/ZHX2/VPS4B/SNX9/CD2AP/RNASE3/TRAF2/DFFA/CARHSP1/PSME1/PSME2/PARK7/PSMD9/SIRT2/RILP/BECN1/DDI2/RIDA/UBXN1/DNAJB2/CSNK2A1/DXO/ATXN3/LARP1/USP25 |
| BP | Nucleobase-containing compound catabolic process | 0.03197444 | 15 | HK2/FUS/RNASEH2A/ZHX2/RNASE3/TRAF2/DFFA/PKLR/DNPH1/CARHSP1/NUDT5/RIDA/DXO/AMPD3/LARP1 |
| BP | Nucleic acid catabolic process | 0.04023286 | 10 | FUS/RNASEH2A/ZHX2/RNASE3/TRAF2/DFFA/CARHSP1/RIDA/DXO/LARP1 |
| BP | Regulation of proteolysis involved in protein catabolic process | 0.04023286 | 10 | CTSC/PSME1/PSME2/PARK7/SIRT2/UBXN1/DNAJB2/CSNK2A1/ATXN3/USP25 |
| CC | Intracellular protein-containing complex | 0.03359358 | 16 | CEP164/RNASEH2A/TRAF2/AKT1S1/PSME1/PSME2/PSMD9/UBAC1/PRR5/EIF4E/DNAJB2/CSNK2A1/PRKAR2A/CENPF/LARP1/DNAJC9 |
| MF | Hydrolase activity | 0.02604439 | 44 | CTSC/CASP1/ILKAP/IMPA1/GLB1/RIGI/ARSB/INPP1/DDX39A/GIMAP7/RNASEH2A/MORC3/SSH3/VPS4B/TGM2/THOP1/RNASE3/TANK/DNPH1/LHPP/STAMBP/NUDT5/CA2/PARK7/ATG4A/AARSD1/PPME1/SIRT2/HAGH/DNAJC6/INPP5D/ASRGL1/GET3/ACP1/DDI2/RIDA/ACYP1/DXO/AMPD3/ATXN3/ERI1/THTPA/USP25/HDDC2 |
| MF | Hydrolase activity, acting on ester bonds | 0.02977448 | 19 | ILKAP/IMPA1/ARSB/INPP1/RNASEH2A/SSH3/RNASE3/LHPP/CA2/AARSD1/PPME1/HAGH/DNAJC6/INPP5D/ACP1/RIDA/DXO/ERI1/HDDC2 |
| MF | mRNA binding | 0.04499447 | 9 | FUS/DDX39A/CARHSP1/PARK7/EIF2S2/EIF4E/RIDA/DXO/LARP1 |

BP: biological process CC: cellular component MF: molecular function

**Supplemental Table 9** Individual metabolites associated with cardiac structure and function in the contemporaneous analysis

| **Echocardiographic measure** | **Compound** | **Class** | **Beta** | **P value** | **FDR** |
| --- | --- | --- | --- | --- | --- |
| LA volume index | Perfluorooctanesulfonic acid (PFOS) | Perfluorochemicals | 1.433854 | 0.000003 | 0.002093 |
| LVEF | Alanine; Sarcosine | Amino acids and derivatives | -1.056170 | 0.000141 | 0.018443 |
| LVEF | Acetylasparagine | Amino acids and derivatives | -1.054792 | 0.000003 | 0.000588 |
| LVEF | Aspartic acid | Amino acids and derivatives | -0.918189 | 0.000003 | 0.000588 |
| LVEF | N-Acetylglutamine | Amino acids and derivatives | -0.609754 | 0.000084 | 0.013805 |
| LVEF | Amino(iso)butyric acid; Dimethylglycine | Amino acids and derivatives | 0.894930 | 0.000330 | 0.036055 |
| LVEF | Pro-Gly | Peptides | -0.800183 | 0.000397 | 0.037105 |
| LVEF | Ala-Ala | Peptides | -0.690145 | <0.000001 | 0.000003 |
| Longitudinal strain | Acetylasparagine | Amino acids and derivatives | -0.600815 | 0.000014 | 0.002339 |
| Longitudinal strain | Aspartic acid | Amino acids and derivatives | -0.595887 | 0.000001 | 0.000285 |
| Longitudinal strain | N-Acetylglutamine | Amino acids and derivatives | -0.446326 | 0.000003 | 0.000664 |
| Longitudinal strain | Pro-Gly | Peptides | -0.598751 | 0.000019 | 0.002478 |
| Longitudinal strain | Ala-Ala | Peptides | -0.391459 | 0.000000 | 0.000047 |
| Longitudinal strain | Bilirubin | Porphyrins and analogues | 0.502911 | 0.000183 | 0.018867 |
| Longitudinal strain | TG(57:7) | Triglycerides | -0.514395 | 0.000202 | 0.018867 |

**Supplemental Table 10** Individual metabolites associated with cardiac structure and function in the lagged analysis

| **Echocardiographic measure** | **Compound** | **Class** | **Beta** | **P value** | **FDR** |
| --- | --- | --- | --- | --- | --- |
| LA volume index | DG(36:4) | Diglycerides | -1.823833 | 0.000189 | 0.041248 |
| LA volume index | Perfluorooctanesulfonic acid (PFOS) | Perfluorochemicals | 1.357982 | 0.000132 | 0.041248 |
| LA volume index | PC(38:3;O) | Phosphocholines | 1.890064 | 0.000164 | 0.041248 |
| LV mass index | Aspartic acid | Amino acids and derivatives | 2.909029 | 0.000030 | 0.018705 |
| LV mass index | Acetylasparagine | Amino acids and derivatives | 3.216491 | 0.000057 | 0.018705 |
| LV mass index | Oleamide | Fatty amides | 6.286250 | 0.000133 | 0.029103 |
| LV mass index | Putative Known Oxylipin (C18H36O4) | Oxylipins | -3.477517 | 0.000213 | 0.034823 |
| Longitudinal strain | SM(36:2;O2) | Sphingomyelins | -1.060906 | 0.000016 | 0.010695 |
| Longitudinal strain | 22:1-Glc-Cholesterol | Sterol esters | -1.553265 | 0.000049 | 0.016141 |

**Supplemental Figures**

**Supplemental Figure 1** Study design and analytic approach


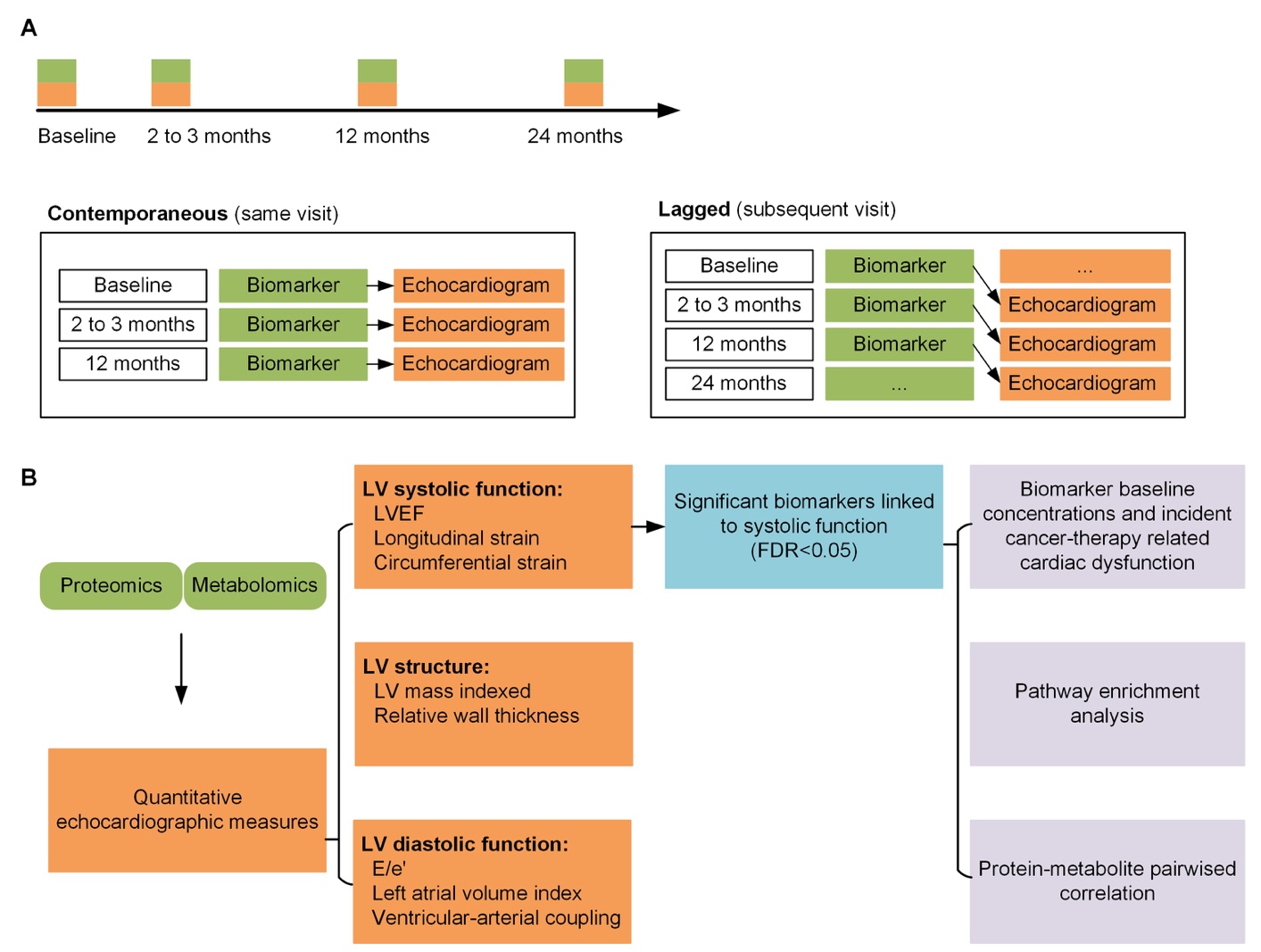


**A.** Blood draw and echocardiography timepoints **B.** Analytic approach for biomarker discovery and pathway enrichment Cancer-therapy related cardiac dysfunction was defined by a ≥10% decline in LVEF to a value <50%.

**Supplemental Figure 2** Time to cardiac dysfunction


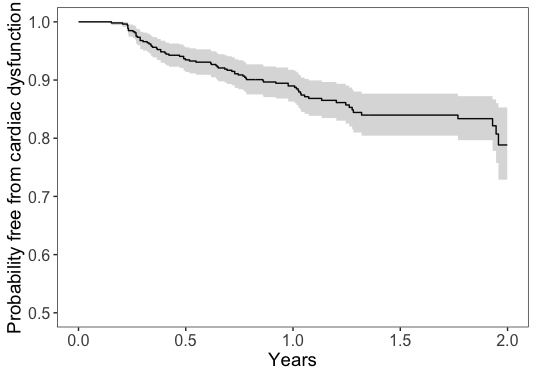


Kaplan-Meier plot showing the overall time to cardiac dysfunction during the follow-up of up to 2 years, as defined by a ≥10% decline in LVEF to a value <50%.

**Supplemental Figure 3** Overlap between the significant associations for proteins with cardiac structure and function in contemporaneous and lagged analysis

**
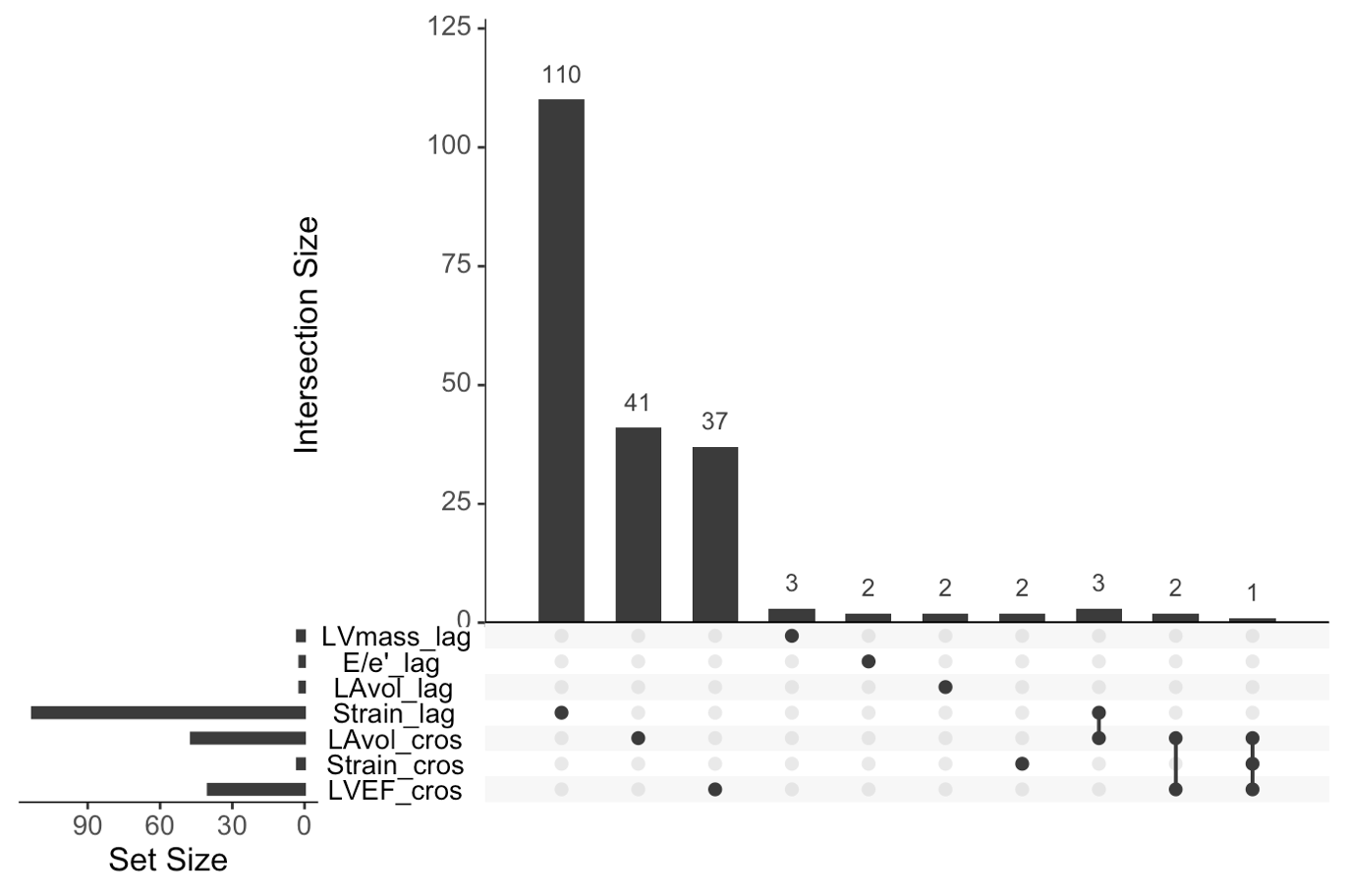
**

Overlap between LA volume index and LVEF in the contemporaneous analysis: Cathepsin C (CTSC), Integrin-linked kinase-associated serine/threonine phosphatase 2C (ILKAP) and GTPase IMAP family member 8 (GIMAP8). Overlap between LA volume index, LVEF, and longitudinal strain in the contemporaneous analysis: Cathepsin C (CTSC). Overlap between LA volume index in the contemporaneous analysis and longitudinal strain in the lagged analysis: myeloid cell nuclear differentiation antigen (MNDA), methylthioribose-1-phosphate isomerase (MRI1), 3'-5' exoribonuclease 1 (ERI1). LVEF: left ventricular ejection fraction; LAvol: left atrial volume index; LVmass: Left ventricular mass index; _lag lagged; _cros cross-sectional

**Supplemental Figure 4** Overlap between the significant associations for metabolites with cardiac structure and function at contemporaneous or lagged analysis


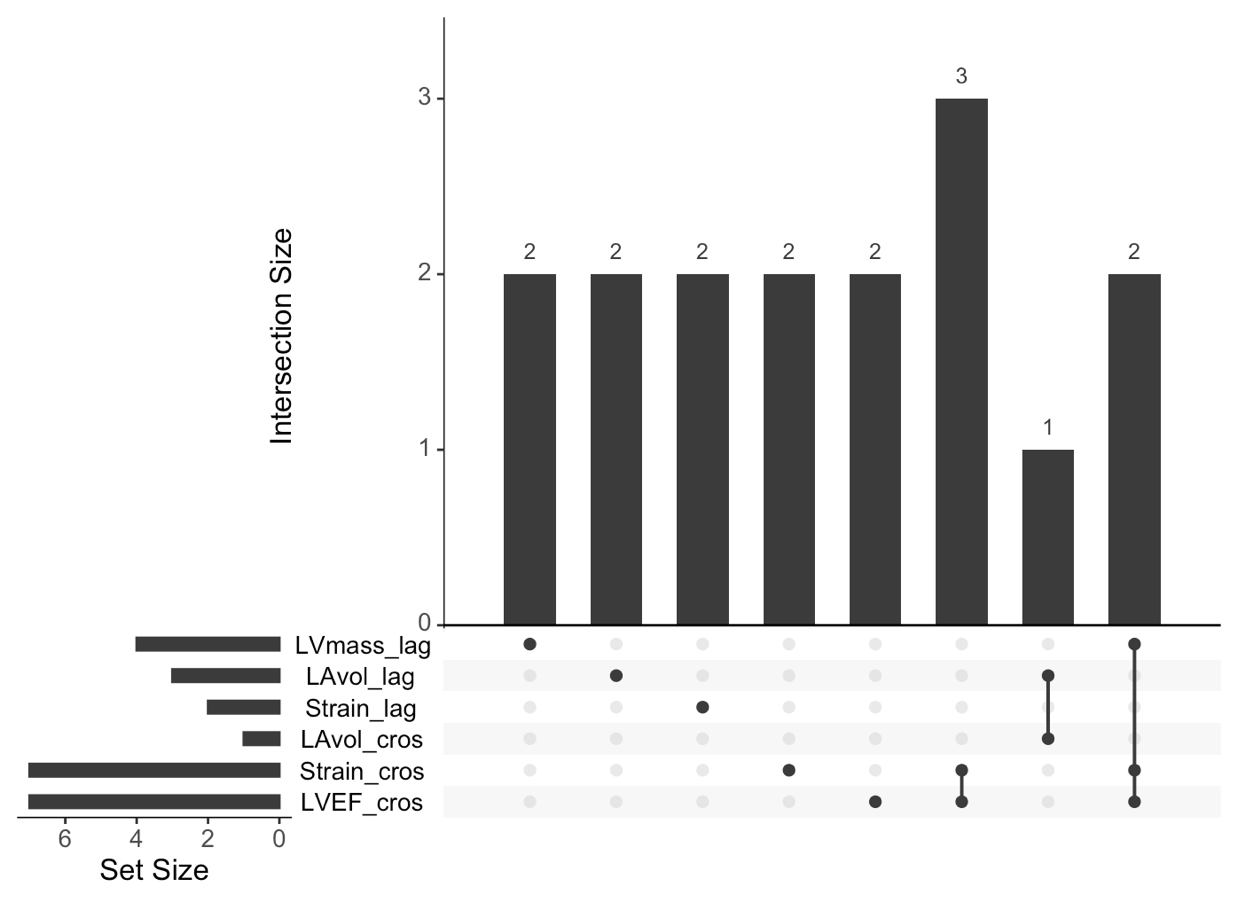


Overlap between LA volume index in the lagged and cross-sectional analyses: perfluorooctanesulfonic acid (PFOS). Overlap between LV mass index in the lagged and longitudinal strain or LVEF in the cross-sectional analyses: aspartic acid, acetylasparagine. LVEF: left ventricular ejection fraction; LAvol: left atrial volume index; LVmass: Left ventricular mass index; _lag lagged; _cros cross-sectional

**Supplemental Figure 5** Correlation between proteins and metabolites that were significantly associated with cardiac function

A
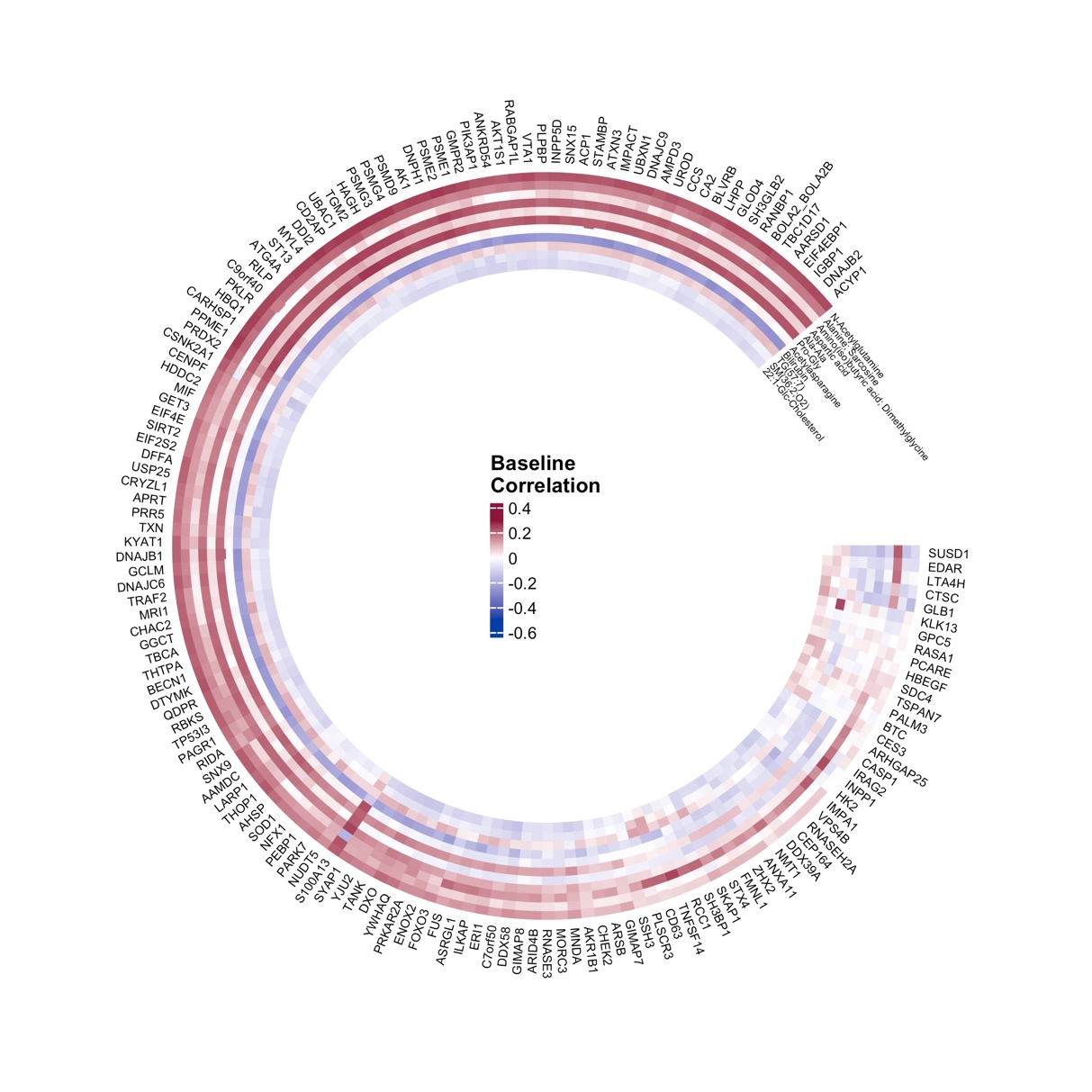


B
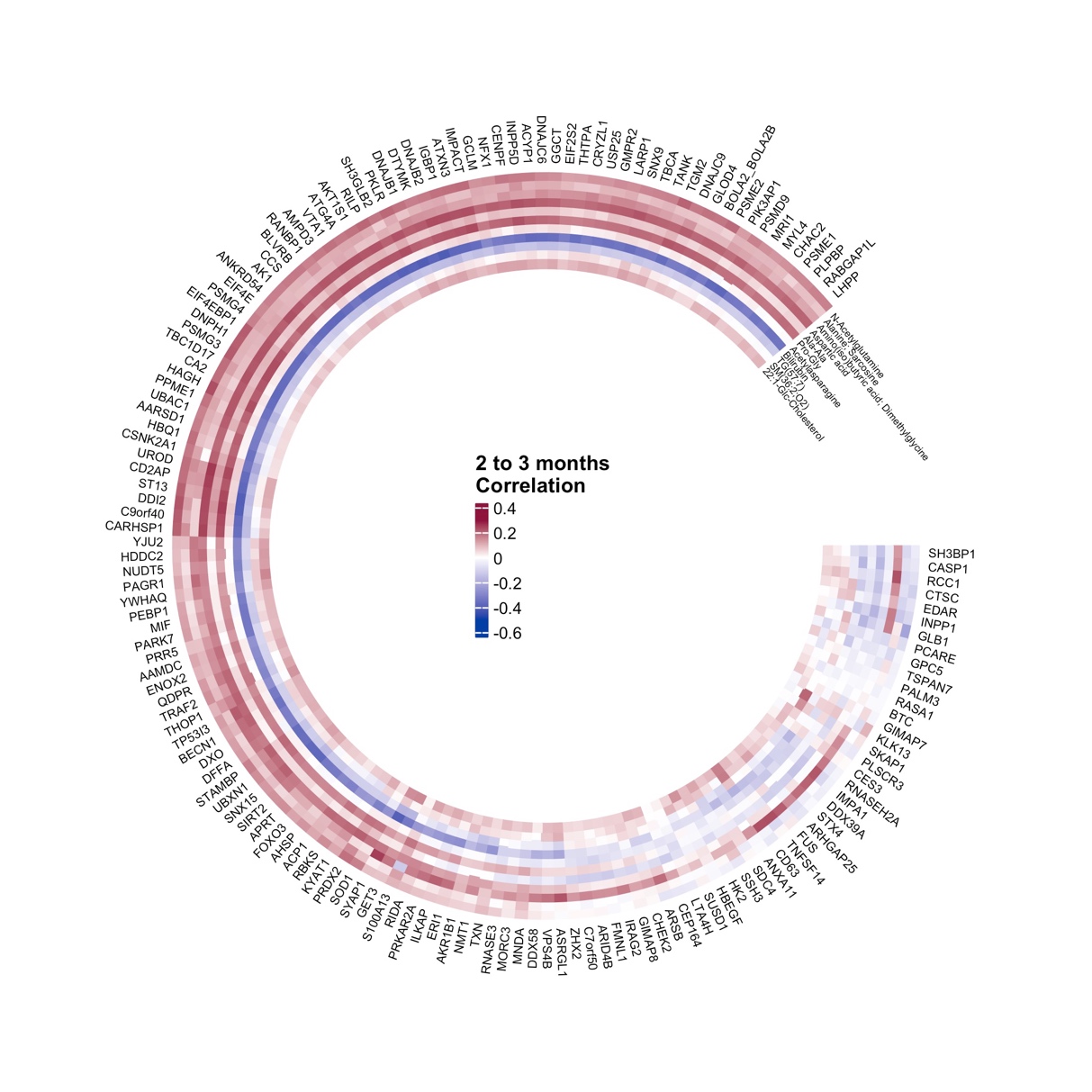


C
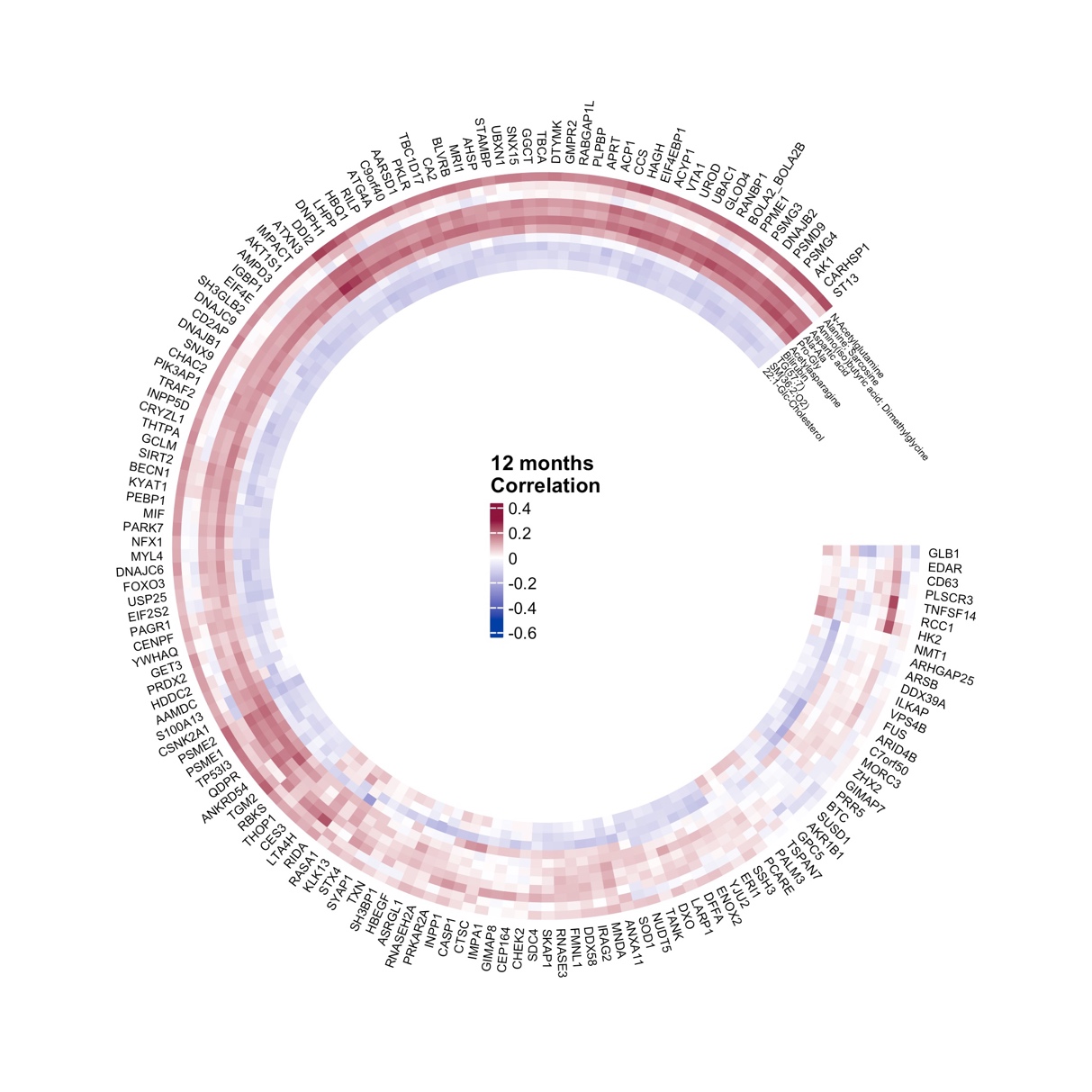


(A) Pairwise correlations between protein-metabolite at baseline, (B) 2 to 3 months, and (C) 12 months were assessed using the Pearson’s method. Significant metabolites are noted in the inner circle (e.g. N-acetylglutamine, and so forth). Significant proteins are noted on the outer circumference (e.g. ST13, and so forth). Red indicates positive correlations between the protein and metabolite, and blue indicates negative correlations, with a darker color indicating a larger absolute value of correlation.
